# Within-Range Translocations and Their Consequences in European Larch

**DOI:** 10.1371/journal.pone.0127516

**Published:** 2015-05-22

**Authors:** Stefanie Wagner, Sascha Liepelt, Sophie Gerber, Rémy J. Petit

**Affiliations:** 1 INRA, UMR1202 BIOGECO, F-33610, Cestas, France; 2 Univ. Bordeaux, BIOGECO, UMR 1202, F-33400, talence, France; 3 University of Marburg, Faculty of Biology, Conservation Biology, D-35032, Marburg, Germany; Instituto de Higiene e Medicina Tropical, PORTUGAL

## Abstract

In contrast to biological invasions, translocations of individuals within a species range are understudied, due to difficulties in systematically detecting them. This results in limited knowledge about the corresponding processes and uncertainties regarding the status of extant populations. European larch, a forest tree whose fragmented native distribution is restricted to the Alps and to other Central European mountains, has been massively planted for at least 300 years. Here we focus on the genetic characterization of translocations having taken place within its native range. Microsatellite variation at 13 nuclear loci and sequence data of two mitochondrial DNA fragments were analyzed on the basis of a comprehensive range-wide population sample. Two complementary methods (Geneclass and Structure) were used to infer translocation events based on nuclear data whereas mitochondrial data were used for validation of these inferences. Using Geneclass, we found translocation events in a majority of populations. Additional cases of translocation and many instances of admixture were identified using Structure, thanks to the clear-cut ancestral genetic structure detected in this species. In particular, a strong divide between Alpine and Central European populations, also apparent at mitochondrial markers, helped uncover details on translocation events and related processes. Translocations and associated admixture events were found to be heterogeneously distributed across the species range, with a particularly high frequency in Central Europe. Furthermore, translocations frequently involved multiple geographic sources, some of which were over-represented. Our study illustrates the importance of range-wide investigations for tracing translocations back to their origins and for revealing some of their consequences. It provides some first clues for developing suitable conservation and management strategies.

## Introduction

Translocations are defined as intentional or accidental anthropogenic movements of living organisms from one place to another ([1], S1 Text). Translocations taking place within a species range have long been performed for agricultural and horticultural purposes and are used increasingly for restocking, reintroduction and assisted migration [2–6]. Like biological invasions, within-range translocations are raising questions on the existence of single versus multiple introduction events, the source of non-native material, and the speed of adaptation to local conditions. However, admixture between native and non-native material is a specificity of within-range translocations with unique evolutionary implications [2, 4, 5]. In principle, translocations can have positive consequences (increased genetic diversity, performance and density of local populations) or detrimental ones (competition, hybridization, genetic swamping and outbreeding depression) [2, 4, 7, 8].

Differentiating native from non-native material can provide critical information for the conservation of genetic resources [2, 9, 10]. Unfortunately, historical information on source and release areas of transferred material is often insufficient or lacking and non-native individuals cannot in most cases be differentiated from native ones by morphological features, especially when admixture between native and non-native material has already taken place [11]. Hence, to date, in spite of their widespread occurrence and increasing frequency across the planet, within-range translocations of undomesticated species have remained understudied [7].

Among the few existing studies, some have focused on admixture between native and non-native stocks of fishes for conservation purposes by relying on Bayesian approaches [12–16]. These studies have demonstrated that restocking can have contrasting consequences, with hybridization rates depending not only on stocking intensity but also on environmental factors. Forest trees have also been the object of a few investigations that focussed on tracing non-native material introduced into native populations [17–19]. These studies, relying on maternally inherited organelle markers, have demonstrate that where translocations had taken place, genetic diversity had increased and genetic differentiation and spatial genetic structure had decreased [18]. They also illustrate that it is not possible to identify *a priori* a native reference sample completely exempt of translocations, as forest history cannot in most cases be reconstructed in detail at the individual stand level [5, 10, 20]. To date, however, no study has explored translocations from a range-wide perspective using a systematic approach.

European forest trees represent particularly good models to investigate within-range translocations. Historical records indicate that forest reproductive material (typically seed or seedlings) has been traded across trees’ native ranges for centuries. However, the overall impact is not clear, as native material might or not be better adapted than non-native one. Moreover, the onset of massive plantations is often quite recent and admixture proceeds on a rather long time scale due to the long generation times of trees [5, 9, 10, 20–22]. Among trees, European larch (*Larix decidua* Mill.) is a good candidate to study within-range translocations due to its restricted pollen dispersal ability that should result in high population structure [23, 24]. Its current fragmented native distribution range was reached in the early Holocene [25, 26], which must also have favoured the build-up of a distinct genetic structure that should help identify translocations. *Larix decidua* is found at high altitudes in the Alps and in other Central European mountains (Carpathians, Sudetes) as well as in the Polish lowlands (collectively referred to as Central Europe; see S1 Fig). In Central Europe, population sizes are rather small due to a particularly pronounced fragmentation in this part of the range. According to historical documents, plantations of larch in Europe have been performed since the sixteenth century and have culminated in the nineteenth century [27]. They have reached such a popularity that foresters used to speak of a “*Lärchenmanie*” (German) or a “*manie du mélèze*” (French) [27–31]. Reports indicate that seed sources from the Tyrol (Austrian and Italian Central Alps) and the Sudetes (mountains at the border between Germany, Czech Republic and Poland) have been used preferentially [29]. A consequence of these widespread translocations was the concomitant expansion of larch canker that contributed to the decline of larch plantations during the twentieth century. The outbreak of this disease has been attributed to the use of maladapted canker-sensitive Alpine material in afforestation (e.g. [32]). In contrast, material from Central Europe, especially from the Sudetes Mountains, is known to be particularly canker-resistant.

The present study focuses on the following questions: Can translocations be accurately identified in *L*. *decidua*, to the point that we can safely reconstruct the ancient genetic structure and identify native material? Are translocations more frequently detected in some parts of the range than in others? Have some seed sources been overrepresented among the transferred material? Have multiple geographic sources been used in plantations? Have translocations been followed by admixture? To answer these questions, we rely on molecular markers and population genetic methods (Geneclass and Structure [33–35]) to systematically detect translocation and admixture events. We establish a reference of native genotypes, compare the ancient genetic structure with the extant genetic structure, and study the intensity, distribution and sources of translocations.

## Material and Methods

### Plant material and DNA isolation

Ascertaining the extent of the native range of *Larix decidua*, in particular in Central Europe, is difficult due to its long plantation history. With permission of the Nordwestdeutsche Forstliche Versuchsanstalt (NW-FVA), we relied on a comprehensive collection of populations sampled in 1957–1958 within the native range to establish an international provenance trial (IUFRO, [27, 32, 36]). Although collecting material *ex situ* rather than *in situ* can add error sources such as seed lot contamination, this particular collection seemed very valuable for our study as it is based on a precise compilation of historical information about the species native range and on large seed samples that should be representative of the original stands [27, 29, 32, 36, 37]. Moreover, some populations sampled at the time might have become eradicated or intermixed with exotic plantations during the more than 50 years that have elapsed since the establishment of this reference collection. In 2010, we collected 32 populations from this trial, including three populations originating from the Swietokrzyskie Mountains and from south of Warsaw in Poland that correspond to the *polonica* variety [38] listed as vulnerable according to IUCN standards (S1 Table, S1 Fig). To improve the coverage of the species native range, we collected eight other populations *in situ*. For comparison, we sampled five populations from beyond the native range as well as one *L*. *sibirica* population from a Swedish provenance trial. In each population, phloem or needles were sampled from 24 individuals. Sampling technique and DNA isolation was as described before [39].

### Microsatellite genotyping

We genotyped 24 individuals per population at 13 highly variable microsatellite loci combined in two multiplexes [39]. Genotype scoring and allele binning based on raw allele sizes had been described before [40]. No correction for null alleles was performed as they had been shown to be rare [39].

### Mitochondrial DNA sequencing

We sequenced two mtDNA regions (UBC460 and *atp*A) from eight individuals per population. Primer sequences for UBC460 are provided in [41] while primer sequences for *atp*A (*L3atp-2-1* 5’- GCGGCTGCCTATAGATACGA-3’ and *L3atpf1/1* 5’- GCTACCGAGGCAGATATGGA-3’) are based on published sequences [42]. PCR reactions were performed in a volume of 25 μl with 1× PCR buffer, 0.24 mM of each dNTP, 0.16 mg/ml of BSA, 0.2 μM of each primer, 1 U of Taq polymerase (DreamTaq Green, Fermentas) and 20 ng of genomic DNA. PCR programs started with an initial denaturation step at 94°C for 4 min followed by 30 (UBC460) or 28 (*atp*A) cycles of denaturation at 94°C for 1 min, annealing at 65°C (UBC460) or 57°C (*atp*A) for 45 s and elongation at 72°C for 2 min 50 s (UBC460) or 1 min 50 s (*atp*A). The cycles were followed by a final elongation step at 72°C for 10 min. PCR products were sent to LGC Genomics (Berlin, Germany) for clean-up and sequencing. Sequence editing and alignment were performed using CodonCode Aligner Version 4.0.3. Haplotypes were detected after concatenating both sequenced regions and their relative frequencies were calculated using GenAlEx 6.41 [43]. For reconstructing minimum spanning networks, we focused on the variable sites, counting each indel as a single event, regardless of its size. The *atp*A region contained a minisatellite with two different repeat motifs and a variable number of repeats. This variation was coded into single nucleotide positions to compute a minimum spanning network using TCS 1.21 [44] with a fixed 12-step connection limit.

### Recent migrant detection

The software Geneclass [33] has been designed to identify recent migrants using nuclear multilocus genotypes. It derives the likelihood of each individual to belong to the population in which it has been sampled. We used it to identify translocation events based on microsatellite genotypes, thereby assuming that non-native individuals occur at low frequency within native populations and that admixture has remained limited. We used simulations and a resampling method [45] to generate 10,000 individuals based on allele frequency distribution for each population. Type I error to falsely identify an individual as a migrant was set at 0.01. A stepwise approach was used to improve detection rates in populations harbouring multiple migrants. The procedure was stopped after three successive runs to keep type I error low.

### Nuclear cluster analysis

Bayesian cluster analysis relied on Structure 2.1 [34, 35]. The number of clusters (*K*) was set from one to nine, with 10 runs for each *K* value. We used a burn-in of 200,000 and 1,000,000 iterations and explored both the no-admixture and the admixture model. To determine the most likely value of *K*, we computed the posterior probabilities and the second order rate of change L”(K) with Structure Harvester [46, 47]. We studied the genetic distances between clusters by deriving a neighbour joining tree from the pairwise distance matrix of the STRUCTURE output using the *R* package APE [48]. In addition, we ran a Principle Coordinate Analysis [43] to identify major genetic groups independently.

### Detection of translocation and admixture events

In each population, we consider as resulting from translocation events all purebred individuals (i.e. individuals assigned to one ancestral group at a given threshold score based on the Structure results) that do not belong to the most frequent ancestral group. By so doing, we assume that (i) prior to translocations a strong ancestral genetic structure existed, with no or limited admixture before the onset of translocations by humans, that (ii) native individuals of each population originate from a single ancestral group, and that (iii) native individuals still represent the majority of individuals. We used simulations to study risks associated with that procedure.

As a starting point to work on genetic exchange, we decided to focus on only two genetic groups, those corresponding to the first axis of the Principal Coordinate Analysis, to accurately detect translocation and admixture events. To confirm our expectation that these two cluster groups are sufficiently differentiated for this purpose, we simulated crosses within the corresponding cluster groups using Hybridlab [49]. The simulated individuals were analysed by Structure and then assigned back to the two cluster groups to evaluate assignment errors. Two different assignment thresholds were explored (0.875 and 0.9375, for details see S2 Fig). In both cases, a selection of the original genotypes was used as learning samples (USEPOPINFO option) and the simulated genotypes were included as supplementary genotypes, i.e. they were not part of the analysis.

### Cytonuclear association

We studied cytonuclear association to investigate if the two cluster groups have only recently become in contact, which would point to human-induced translocations. The rationale is that, if two populations that initially differ at both organelle and nuclear genomes come into contact, individuals exhibiting organelle genomes of one population and nuclear genomes typical for the other population will be generated within a few generations through crossbreeding, assuming selective neutrality. Conversely, if not all possible cytonuclear combinations are detected in a given population, very recent human-induced mixing can be inferred.

### Reconstruction of the ancestral genetic structure

We combined data from Geneclass and Structure to identify all individuals that are not fully native (recent migrants identified by Geneclass and non-native purebred and admixed individuals identified by Structure). We then compared summary statistics before and after excluding these individuals, focusing on allelic richness *N*_A_, gene diversity *H*_S_, inbreeding coefficient *F*_IS_ and fixation index *F*_ST_ measured using FSTAT version 2.9.3.2 [50] with 15,000 permutations. For mtDNA data, we compared the diversity index *h*_S_ and the coefficient of genetic differentiation *G*_ST_ [51] using Permut (http://www.pierroton.inra.fr/genetics/labo/Software/).

### Patterns of translocation

To study if the two regions (Alps and Central Europe) had experienced asymmetric exchanges of genetic material, we compared the proportion of translocation events identified with Structure in each region using a *z*-test. Then, to investigate the origin of the material used for translocation, we compared the genetic composition of non-native individuals (recent migrants detected by Geneclass and non-native purebreds detected by Structure) and native individuals (all remaining purebreds). In this analysis, to avoid including false positives, admixed individuals identified by Structure were not included unless they had been identified by Geneclass as being recent migrants. Finally, to study if translocations had involved multiple geographic sources, we counted the number of genetic clusters identified by Structure in populations with versus without evidence for translocation. An individual was considered to belong to a given nuclear cluster if its ancestry was mainly found in this cluster (assignment value >0.5). In populations without evidence for translocation, we considered all clusters except one (the native one); in populations with evidence for translocation, we considered all clusters except two, the native and the most frequent non-native one. We used a Wilcoxon rank-sum test based on the test statistic *W* to compare the mean number of extra-clusters between these two groups of populations [52].

### Patterns of admixture

First, we compared the proportion of admixture events identified with Structure in the Alpine versus the Central European region using a *z*-test. Second, we tested if translocations eventually result in admixture using two different approaches. In a first approach, we predicted that the proportion of admixed individuals should be higher in populations in which translocation events have been detected than in the remaining populations, as contact between populations necessarily precedes admixture. To test this expectation, populations were grouped in two categories: populations with evidence for translocation and populations without such evidence, as explained above. Mean admixture level was then compared between these categories (after excluding individuals that had provided evidence for translocation), using a one-sided *t*-test. In a second approach, we relied on mitochondrial data to study if translocations eventually result in admixture. As individuals of non-native origin should typically be present at low frequency and will then be more likely to mate with local native individuals, whereas local native individuals should mostly mate with each other, the proportion of individuals with admixed genomes should be higher among individuals exhibiting non-native mitochondrial haplotypes than among individuals possessing native haplotypes. These two proportions were measured in each region (Alps and Central Europe) and compared using a *z*-test.

## Results

### Microsatellite genotyping and mitochondrial DNA sequencing

**Fig 1:**
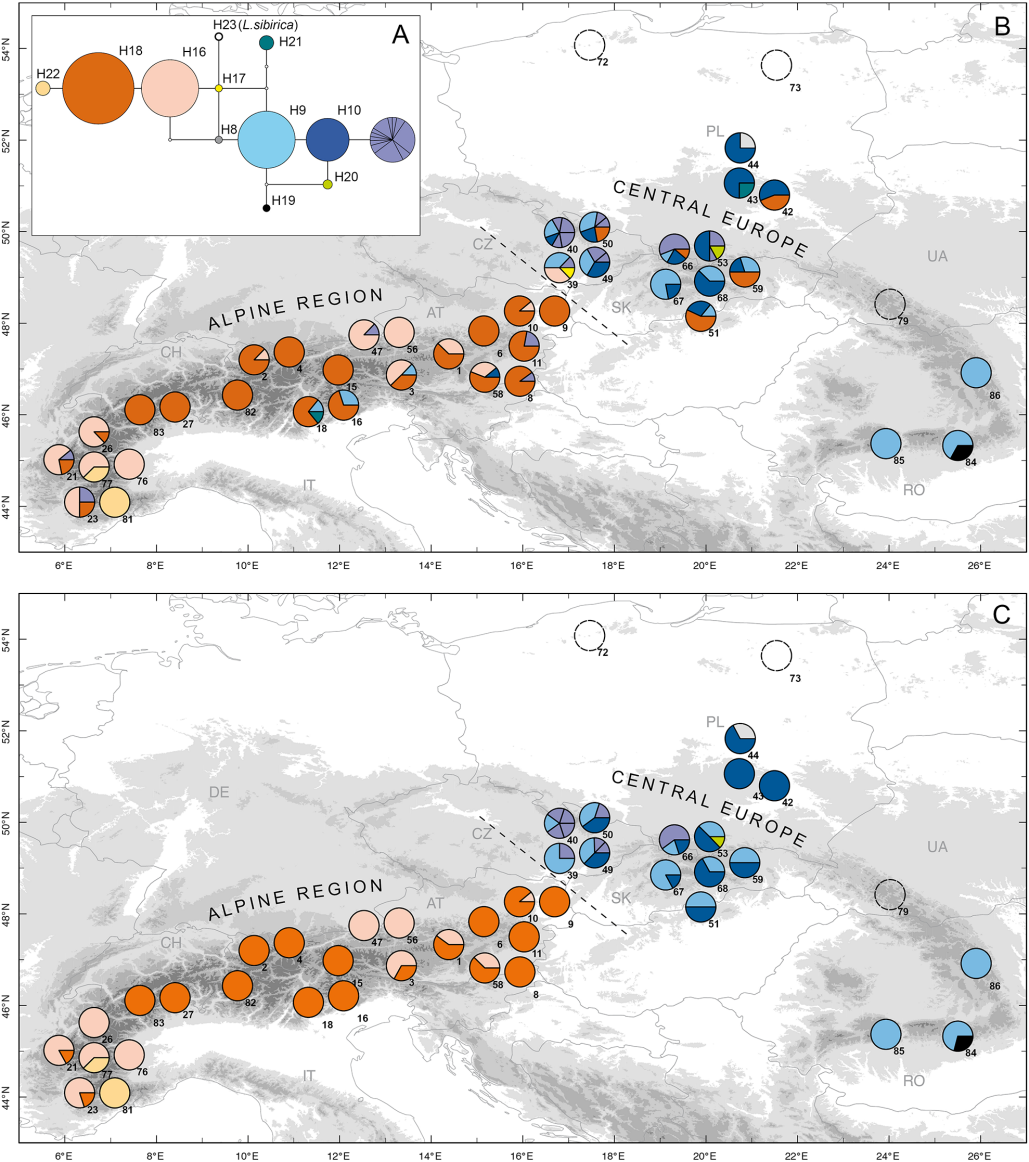
Results of mitochondrial analyses. Minimum spanning network (A), distribution of combined mitochondrial haplotypes before (B) and after (C) translocation removal. In (A) circles represent haplotypes colored by their codes and scaled to their frequencies. Haplotypes caused by minisatellite variation are in purple. White circles correspond to predicted haplotypes that were not observed. Branches correspond to single mutations regardless of their length. In (B) and (C) circles represent the haplotype composition of the 40 presumably native populations and three populations sampled outside the range (n°72, 73 and 79) (~8 individuals/population).

A sample of 1026 *Larix decidua* individuals from 45 populations were successfully genotyped at 13 microsatellite loci. We detected on average 22 alleles per locus. Sequencing of the two mtDNA fragments in 363 individuals from 43 populations led to the detection of 22 mtDNA haplotypes. The observed molecular variation was a combination of nucleotide substitutions, insertions/deletions as well as copy number variation of the minisatellite (S2 Table). To illustrate the relationships between haplotypes we constructed a minimum spanning network (Fig 1A). The eight *Larix sibirica* samples are characterized by a single private haplotype (H23) that has a central position in the network. The *L*.*decidua* haplotypes form two groups (i.e. lineages), each composed of two frequent haplotypes and some less frequent ones. In lineage 1 (H16, H17, H18 and H22), haplotypes H16 and H18 were found in 58% of the individuals. In lineage 2 (H8, H9, H10, H19, H20, H21 and 12 additional rare haplotypes differing in copy number of one minisatellite motif), haplotypes H9 and H10 occurred in 28% of the individuals (Fig 1B).

### Recent migrant detection

Three nested runs of Geneclass led to the detection of 79 (9%) recent migrants in 33 of the 40 native populations (S3 Fig). Many populations were characterized by one migrant (10 populations), two migrants (9 populations) or three migrants (8 populations). In addition, there were three populations with four migrants and another three with five migrants. In seven populations no migrant was detected.

### Bayesian cluster analyses

**Fig 2:**
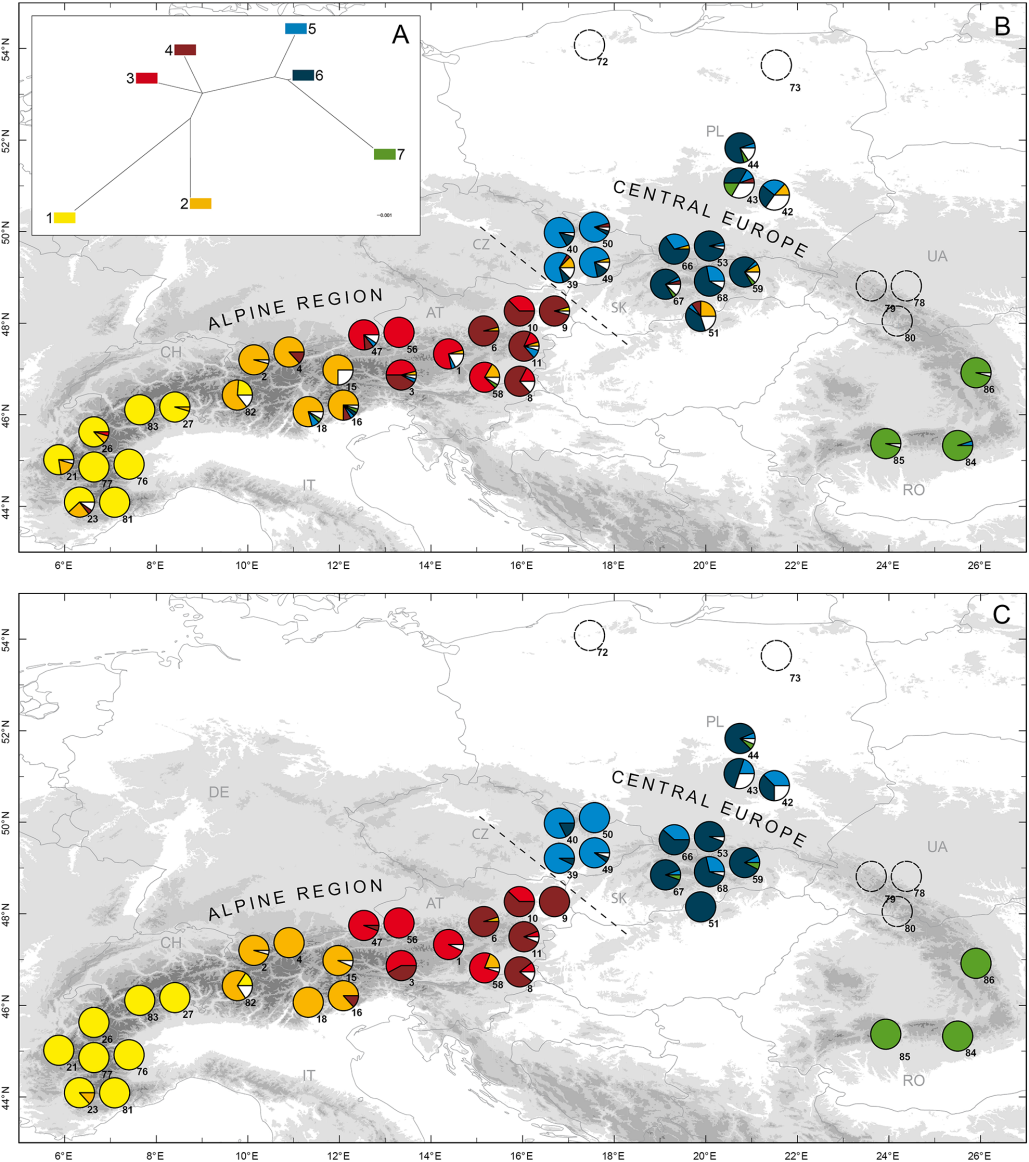
Nuclear clusters detected by Structure. Neighbor joining tree (A) and distribution of the seven SSR clusters before (B) and after (C) removing presumably non-native genotypes. In (A) each rectangle represents a cluster. In (B) and (C) pie charts represent cluster composition of the 40 presumably native populations and 5 populations sampled outside the range (n°72, 73, 78, 79 and 80) (~24 individuals/population). Individuals with *q* values > 0.5 are colored, the remaining ones are in white.

**Fig 3:**
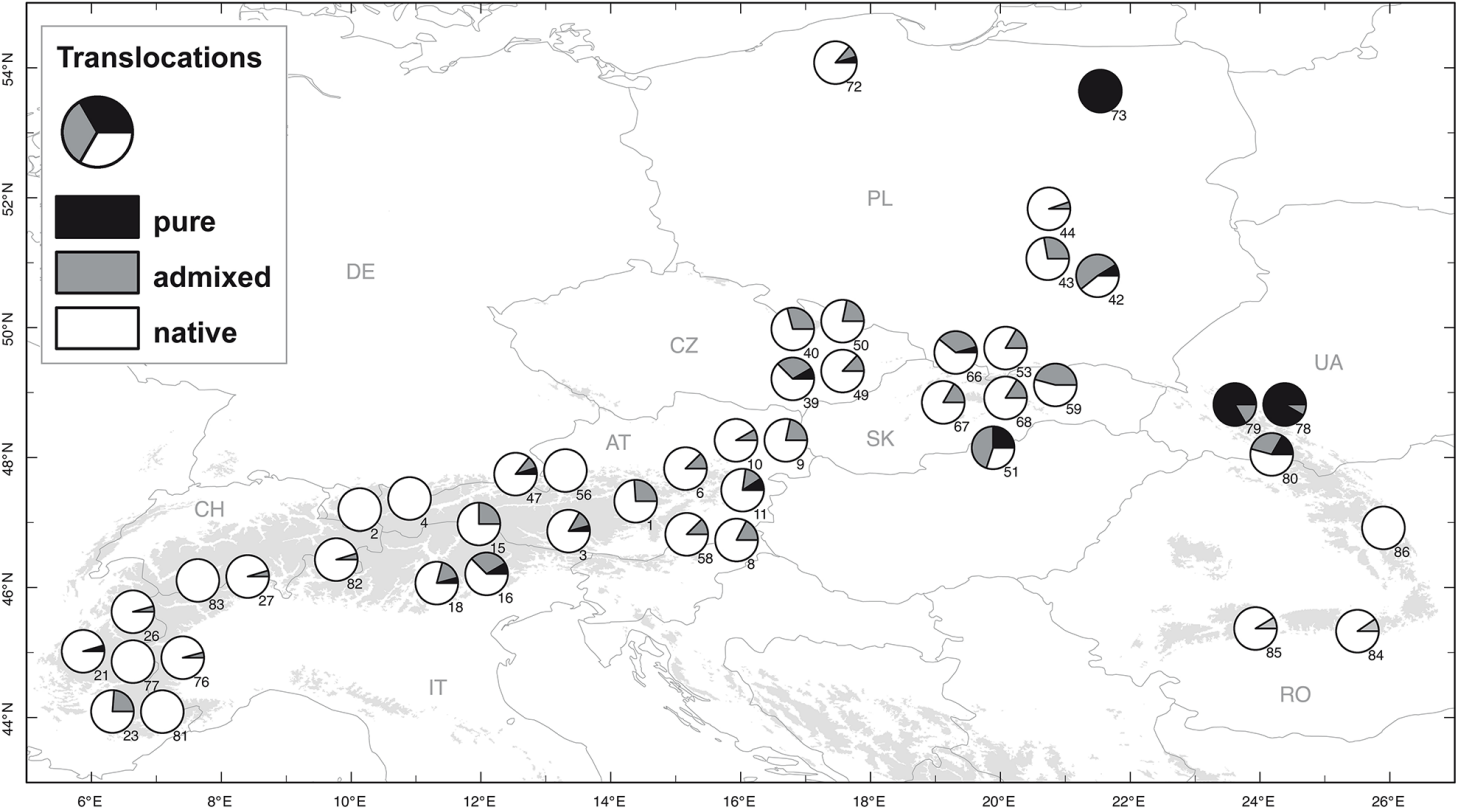
Non-native purebreds and admixture events detected with Structure. Populations sampled within the native range and five populations sampled outside the native range (n°72, 73, 78, 79 and 80).

Cluster analyses of the 1026 *Larix decidua* genotypes revealed support for the existence of three ancestral groups (*K* = 3) using the model without admixture (S4 Fig). The model with admixture identified a similar solution at *K* = 3 but detected another solution at *K* = 7 supported by the posterior probabilities and the second order rate of change (S5 Fig, Fig 2A and 2B). This latter solution identifies clusters that are hierarchically nested within the three larger clusters. We selected the solution at *K* = 7, as it provides increased resolution when reconstructing the ancestral genetic structure across the species range. To study translocations, we grouped the seven clusters into two particularly strongly differentiated groups: group 1 made of clusters 1–4 (corresponding to clusters 1+2 in the solution at *K* = 3, these two clusters being the most closely related) and group 2 made of clusters 5–7 (corresponding to cluster 3 in the solution at *K* = 3). These two groups were also distinguished on the first axis of the principle coordinate analysis, which explains 38% of the variation, compared to 23% for the second axis (S6 Fig). Simulations confirmed that assignment power is sufficient to differentiate between these two cluster groups. The two thresholds used to assign genotypes into purebred and admixed category led to similar inferences, so we relied on the more relaxed threshold (0.875) (S3 Table,). When simulating crosses between individuals belonging to the same cluster group, the resulting genotypes were never falsely assigned to the other cluster group. Moreover, the risk to falsely assign them to the admixed category was quite low: the rate of false positives was 13.3% of the simulated individuals in group 1 and 12.3% in group 2; even lower rates were observed under a more realistic scenario (S3 Table). These results confirm that assignment power is sufficient to differentiate between individuals originating from the two cluster groups (clusters 1–4: group 1; and 5–7: group 2). Applying the assignment threshold of ≥0.875 for each of the two cluster groups on the 40 populations, we detected 18 (2%) purebred individuals in populations otherwise dominated by the other cluster group (Fig 3). These 18 individuals, distributed in six Alpine and four Central European populations, were considered to represent recent translocation events. In five populations, one individual considered to result from translocation was detected, in four populations two such individuals were detected and in one population (population 51) as many as five individuals were detected. We also found 141 admixed individuals (16%) in 32 of the 40 populations (Fig 3).

### Cytonuclear analysis

We checked the association between nuclear assignments and mitochondrial lineages for all studied individuals sampled within the range that had been studied with both markers. We found 15 out of 40 populations harbouring both mitochondrial lineages. These 15 populations included the ten populations within which we had also detected both types of nuclear purebreds. Yet, at the individual level, all of the 166 nuclear genotypes assigned to group 1 purebreds carried mtDNA haplotypes from lineage 1, and all but one of the 97 group 2 purebred individual carried mtDNA haplotypes from lineage 2, indicating nearly total cytonuclear association (Table 1). This suggests that the contact between these divergent genetic groups is recent, thereby supporting our conclusion that individuals assigned to the locally uncommon nuclear group or mitochondrial lineage are not native.

**Table 1:** Counts of mito-nuclear genotype combinations found in 40 populations.

		nuclear group 1 purebred	nuclear group 2 purebred	admixed	total
**All (Alps +**	**mt lineage 1**	166	1	37	204
**Central Europe)**	**mt lineage 2**	0	96	36	132
**Alps**	**mt lineage 1**	160	0	24	184
	**mt lineage 2**	0	7	7	14
**Central Europe**	**mt lineage 1**	6	1	13	20
	**mt lineage 2**	0	89	29	118

### Reconstruction of the ancestral genetic structure

By combining results from both Geneclass and Structure, we detected 208 (23%) presumably non-native individuals distributed in 38 of the 40 populations. There were only 30 individuals identified by both methods (six purebred and 24 admixed individuals). In particular, using Geneclass, we identified 49 migrants not detected with Structure. As expected, Geneclass was less efficient at detecting migrants in populations characterized by high rates of admixture (S7 Fig). Geographic structure obtained after removing these 208 individuals is shown in Figs 1C and 2C. For nuclear data, global summary statistics before and after removing these individuals differed significantly for all measures, with the trimmed dataset having lower within population diversity, increased differentiation among populations and being closer to Hardy-Weinberg equilibrium than the original dataset (*N*_A_ = 6.2 vs. 6.6, *H*_S_ = 0.74 vs. 0.76, *F*_ST_ = 0.11 vs. 0.08, *F*_IS_ = 0.02 vs. 0.05; *p* < 0.001 in all cases). Note however that a few populations still had comparatively high heterozygote deficit after removing individuals considered to be non-native (*F*_IS_ > 0.08 for populations 3, 53, 66 and 84, S4 Table,). Genetic diversity at mtDNA markers was also lower and genetic structure higher in the trimmed dataset (*h*_S_ = 0.25, *G*_ST_ = 0.67) than in the original one (*h*_S_ = 0.37, *G*_ST_ = 0.52). In fact, after trimming, most populations became dominated by a single genotypic cluster. Two large regions of the range, the Alps and Central Europe, turned out to be composed of individuals characterized by reciprocally exclusive nuclear clusters and mitochondrial lineages: all populations of the Alpine region were dominated by individuals with nuclear genotypes assigned to cluster group 1 and characterized by mitochondrial lineage 1, and all populations from Central Europe were dominated by individuals with nuclear genotypes assigned to cluster group 2 and characterized by mitochondrial lineage 2, making it possible to compare in detail the translocations across these two regions.

### Patterns of translocation

We found evidence for translocation in larch populations collected both *in situ* and *ex situ* in provenance tests, indicating that the collection mode did not strongly influence results. The proportion of individuals considered to originate from translocation events was not significantly different in the Alpine and in the Central European region (2% versus 3%, *z* = -1.4, *p* = 0.17). Considering results from both Geneclass and Structure, we found that one of the four Alpine clusters (cluster 2) was over-represented in non-native material from Central Europe (50%; S8 Fig). This cluster is made up of individuals from populations of the Austrian and Italian Central Alps, including from the Tyrol (S1 Fig). Genotypes with this cluster were also overrepresented when examining translocations within the Alpine region (S8 Fig). In contrast, none of the three Central European clusters was over-represented when considering translocations from Central Europe to the Alps or within Central Europe. To check if each population typically harbours translocations from a single source or from several ones, we focussed on the Alpine region where more populations had been sampled and where the geographic structure is stronger. We found a mean of 1.8 non-native additional clusters in the six populations with evidence for translocation, compared to only 0.8 in the 18 populations without evidence for translocation. This difference is significant (*W* > 148, *p* < 0.001), suggesting that when translocations occur in a given population, they often involve material from more than one source.

### Patterns of admixture

**Fig 4:**
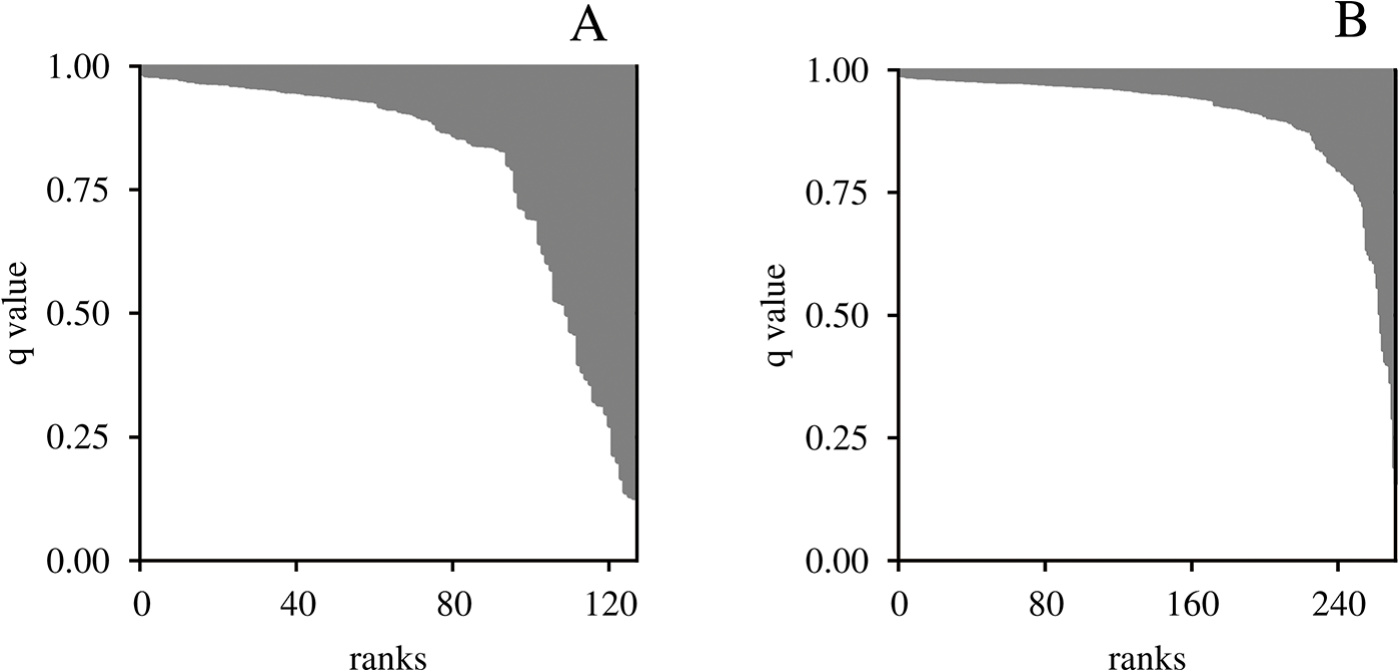
Admixture in Central European populations. Populations with evidence for recent translocation (A) and without evidence (B). Bars represent individual *q*-values (group 2 in white, group 1 in grey).

The proportion of admixed individuals was significantly lower in Alpine populations than in Central European ones (10% versus 23%, *z* = -1.5, *p* < 10^–5^). Furthermore, mean admixture values were significantly higher in populations with evidence for translocation than in populations without evidence for translocation (Alps: *q* = 0.08 versus 0.05, *p* < 10^–4^, Central Europe: *q* = 0.19 versus 0.09, *p* < 10^–4^, Fig 4). Finally, admixture levels were higher in individuals harbouring the non-native mitochondrial lineage: in the Alps, 50% (7/14) of the individuals with mtDNA haplotypes from lineage 2 were admixed, compared to only 13% (24/184) for individuals with mtDNA haplotypes from lineage 1 (*z* = -3.7, *p* < 10^–3^). Similarly, in populations from Central Europe, 65% (13/20) of the individuals with mtDNA haplotypes from lineage 1 were admixed compared to only 25% (29/118) for individuals with mtDNA haplotypes from lineage 2 (*z* = -3.6, *p* <10^–3^).

### Genetic composition of populations sampled outside the species range

All five populations were located in Central Europe. Yet 59% of all individuals sampled were made of pure Alpine material, i.e. a much higher proportion than for the 16 reference populations from Central Europe (3%, Fig 3). There were also 15 admixed individuals in these five populations. In the three populations where mitochondrial data was available (populations 72, 73 and 79), strict cytonuclear association was observed, as in the native range, pointing to limited introgression and hence recent plantations.

## Discussion

Selecting suitable methods to detect translocations and investigate their consequences is challenging, as it implies differentiating conspecific individuals that originate from different parts of the range in a context where admixture has already taken place. Yet, this detection task is of fundamental importance to determine the status of extant populations [2, 9, 12, 14, 53, 54]. In this study, we took advantage of several suitable features of *Larix decidua*, including the persistence of native genotypes in all parts of the range and a strong geographic structure at both nuclear and mitochondrial markers.

To systematically detect translocations, we first relied on a classical assignment method to exclude reference populations as the origin of individuals on the basis of multilocus genotype data (Geneclass, [33]). The advantage of this approach is that there is no need to investigate the entire genetic structure of the species. However, in populations where high rates of admixture had been detected, Geneclass was less effective at detecting introduced material, showing that this method also has limitations, even if some admixed individuals were identified as migrants. While recent migrants can in principle occur by natural means, they were conservatively considered to represent non-native material.

The other method used to identify translocations relies on the extant range-wide genetic structure identified using Structure [35]. We showed using simulations (Hybridlab, [49]) that assignment power was sufficient to detect translocations. These translocations could also be validated with mitochondrial data: individual genotypes identified with nuclear DNA data as being non-native were also consistently shown to be non-native using mitochondrial data. Yet only a few generations of crossbreeding would have sufficed to reshuffle mitochondrial lineages and nuclear clusters through hybridization and backcrosses, suggesting that genetic exchanges between the two ancestral genetic groups have been limited. This point supports our assertion that the corresponding individuals found in minority in a population consist indeed mostly of recent long-distance translocation events. The limitation of this second approach is that it depends on our ability to detect a strong ancestral genetic structure. In our study, we conservatively considered only two ancestral groups of populations, to reduce assignment errors, thereby trading power for accuracy. Hence, only a subset of the translocation events could be identified, thus underestimating the frequency of translocations and biasing the results towards the identification of long distance translocation events. This should be kept in mind when interpreting the results.

The two approaches used to detect translocation and admixture events turned out to be complementary as they identified largely non-overlapping sets of presumably non-native individuals. Once all these individuals had been removed, a clear-cut geographic pattern emerged. In particular, all populations from the Alps turned out to be genetically different from Central European populations. Hence, we were not compelled to resort to post hoc arguments to dismiss populations from our reference set. In fact, some populations from Central Europe had a very high proportion of non-native genotypes, and the pre-plantation genetic structure that we inferred is probably still not completely devoid of exotic material, as shown by the persistence of relatively high heterozygote deficit in some populations.

We found that translocations had preferentially involved one of the four Alpine genetic clusters. In the populations sampled outside of the species range, genotypes assigned to this cluster were also particularly frequent. This finding is in agreement with historical information indicating that *L*. *decidua* seeds from the Tyrol had been intensively used in plantations [29]. In contrast, we could not confirm a preferential use for plantations of seeds from the Sudetes, as documented in the literature [26].

Our results also show that translocations had typically involved multiple geographic sources. This result is reminiscent of earlier studies in oaks based on chloroplast DNA, where planted stands were shown to have much greater diversity than native ones, likely as a consequence of the use of mixed seed lots during plantations [55]. Another possible explanation for this observation is that there had been several episodes of plantations at each site, involving different seed sources.

We found strong support for a link between translocation and admixture, as expected if admixture events had taken place subsequently to translocations, once planted trees have become mature. First, we found an increased proportion of admixed individuals in populations where one or more non-native purebred genotypes had been detected, pointing to the recent onset of admixture between native and non-native trees. Second, individuals with non-native mitochondrial haplotypes also had elevated rates of admixture. Although some admixed individuals are likely false positives, the previous findings as well as the high proportion and broad distribution of admixture suggest that in many places at least one generation has elapsed since the first trees were introduced, ruling out seed lot contamination in plantations as the main source of translocation events. The particularly high admixture rate detected in Central Europe makes sense as the distribution of *L*. *decidua* in Central Europe is more fragmented than in the Alps and populations there are of rather small size, making them more susceptible to genetic swamping [4, 5]. Translocations and the associated admixture process uncovered in this study are probably a result of the intensive periods of larch planting during the eighteenth and nineteenth century [27–31]. Hence, our results shed new light on the “*Lärchenmanie*” by uncovering its irreversible long-term consequences through a modification of the genetic structure of the species, including in areas where threatened gene pools have been recognized (*cf*. *L*. *decidua* variety *polonica*). Finally, the results for populations sampled beyond the native distribution range show that we are now in the position to start investigate their make-up and subsequent history.

In conclusion, our study illustrates the importance of investigating translocations across species range to efficiently and accurately trace non-native material of different geographic origins and to uncover native material as well as species ancient history. In the future, such approaches will provide essential information for conservation of genetic resources. For instance, in *L*. *decidua*, we could now in principle identify canker-sensitive Alpine material introduced across the range and native Polish populations threatened by introgression, using greater sample sizes. While further progress should be achieved through the development of new demo-genetic methods that simultaneously identify introductions, admixture events and their timing, existing population genetic tools can already be used more widely, thereby revealing the frequency and prevalence of translocations across the globe as well as their consequences.

## Supporting Information

S1 Fig Current distribution range of *Larix decidua* and studied populations. (DOCX)

S2 Fig Simulation study. (DOCX)

S3 Fig Distribution of migrants detected by Geneclass. (DOCX)

S4 Fig Individual bar plots of Structure model with and without admixture. (XLSX)

S5 Fig Determining the number of populations with Structure using Evanno’s method. (DOCX)

S6 Fig Results of Principle Coordinate Analyses. (DOCX)

S7 Fig Comparison Geneclass and Structure results. (DOCX)

S8 Fig Genetic composition of presumed natural and non-native material. (XLSX)

S1 Table Sampling information of the studied *Larix decidua* populations. (DOCX)

S2 Table Nucleotide and minisatellite variation of 23 mitochondrial haplotypes. (DOCX)

S3 Table False positives detection by simulations. (DOCX)

S4 Table *F*_IS_ before and after removing individuals considered to be non-native. (DOCX)

S1 Text Definitions. (DOCX)
