# Supplementary material for: Within-Range Translocations and Their Consequences in European Larch
Source: PLoS One. 2015 May 22;10(5):e0127516. doi: 10.1371/journal.pone.0127516 (PMC4441476; doi:10.1371/journal.pone.0127516)
Supplement: S1 Fig — (DOCX) [file pone.0127516.s001.docx]

**S1 Fig.** Current distribution range of *Larix decidua* and studied populations. Black dots correspond to native populations, white dots to populations sampled beyond the range. Modern distribution data compiled by E. Welk, AG Chorology, Geobotany Department, University of Halle, based on map 21b in Meusel *et al.* (1965), and ourselves. Map produced using ArcGIS 9.3 (ESRI 2009).


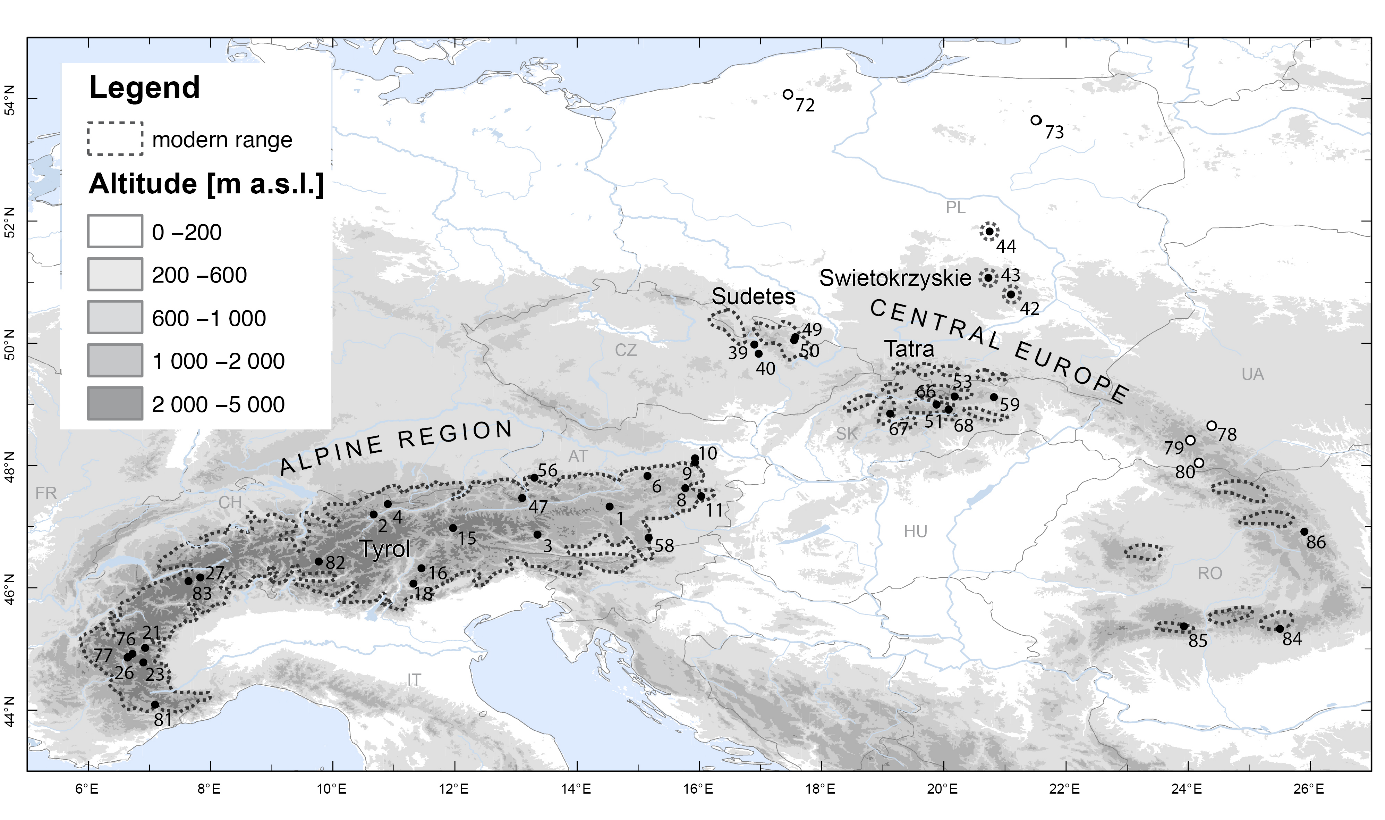


**Reference**

1. Meusel, H., Jäger, E.J., Weinert, E. (1965): Vergleichende Chorologie der zentraleuropäischen Flora. Text u. Karten. Bd. 1. - VEB Fischer Jena.
