# Supplementary material for: Within-Range Translocations and Their Consequences in European Larch
Source: PLoS One. 2015 May 22;10(5):e0127516. doi: 10.1371/journal.pone.0127516 (PMC4441476; doi:10.1371/journal.pone.0127516)
Supplement: S2 Fig — (DOCX) [file pone.0127516.s002.docx]

**S2 Fig. Simulation study**

**
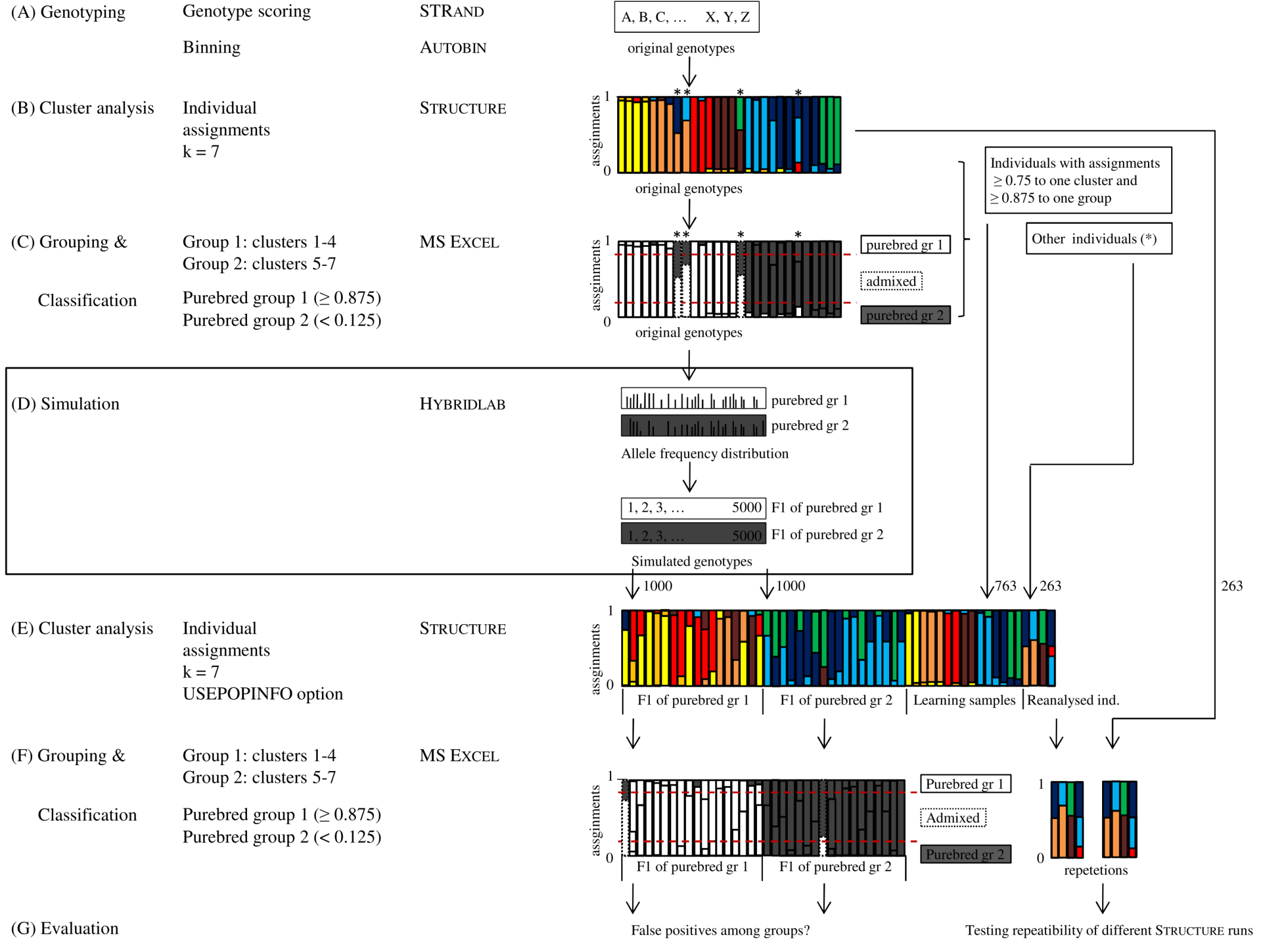
**

We first genotyped the 1026 individuals of the original sample and analysed them using Structure (A, B). We selected the solution at *K*=7, as explained in the text. Assignment scores to clusters 1-4 and to clusters 5-7 were grouped to obtain only two values for each individual genotype (C). This indirect procedure (compared to the procedure consisting of directly assigning genotypes to two populations) was selected because it appeared to be geographically more consistent, taking advantage of the greater resolution at *K*=7. Individuals were then classified into purebred and admixed groups based on the following thresholds: ≥0.875 (purebred 1), 0.125≤x<0.875 (admixed) and <0.125 (purebred 2), corresponding to the optimal theoretical assignment thresholds to distinguish backcross 1 from purebred individuals. (C). Purebreds of each group were selected to provide the basis of the simulation experiment (D). Based on the allele frequency distributions of the two purebred groups we simulated random mating within each group to generate 2×5000 F1 genotypes. A random sample of 2×1000 F1 simulated genotypes (1000 for each purebred group) was analysed using Structure with the parameters of the original analyses, i.e. in particular with *K*=7 (E). To obtain comparable results these 2000 genotypes played no role in the definition of clusters. Instead, the Structure analysis relied on 763 learning samples (USEPOPINFO option). These 763 genotypes correspond to individuals of the original analysis that were assigned to one of the seven clusters with an assignment threshold of ≥0.75 and which were assigned to one of the purebred groups using thresholds ≥0.875 or ≤0.125. The remaining samples of the original sample (263) were reanalysed as supplementary genotypes (like the simulated genotypes) to check if assignments are comparable to those for the same genotypes in the original Structure analysis. Finally, individual assignments (*K*=7) were combined as before to yield two values and genotypes were categorized into purebred and admixed groups using a threshold of 0.875 (F). We then counted the proportion of false positives among simulated F1 genotypes for each group (individuals missassigned as purebreds from the other group or as admixed genotypes) and compared the individual assignments of the remaining 263 original genotypes (USEPOPINFO option) with those for the same genotypes in the original analysis.

The scheme described and illustrated above corresponds to a conservative scenario (inter-cluster scenario). We also tested another, more realistic scenario (intra-cluster scenario) that differs in that we simulated intercrossing within each of the seven clusters based on the allele frequency distribution of each of these clusters. This simulation was repeated but using different thresholds: ≥0.9375 (purebred 1), 0.0625≤x<0.9375 (admixed) and <0.0625 (purebred 2), corresponding to the optimal theoretical thresholds to distinguish backcross 2 from purebred individuals. There were therefore three simulations in total: one corresponding to the inter-cluster scenario and two corresponding to the intra-cluster scenario), where two sets of thresholds were compared.
