# Supplementary material for: Within-Range Translocations and Their Consequences in European Larch
Source: PLoS One. 2015 May 22;10(5):e0127516. doi: 10.1371/journal.pone.0127516 (PMC4441476; doi:10.1371/journal.pone.0127516)
Supplement: S3 Fig — (DOCX) [file pone.0127516.s003.docx]

**S3 Fig.** Distribution of migrants detected by Geneclass. Pie charts represent 40 populations sampled within the native range and five populations sampled outside the native range (n°72, 73, 78, 79 and 80). Spatial classification based on the likelihood calculations implemented in Geneclass and comparison to cluster groups defined by the Structure approach.


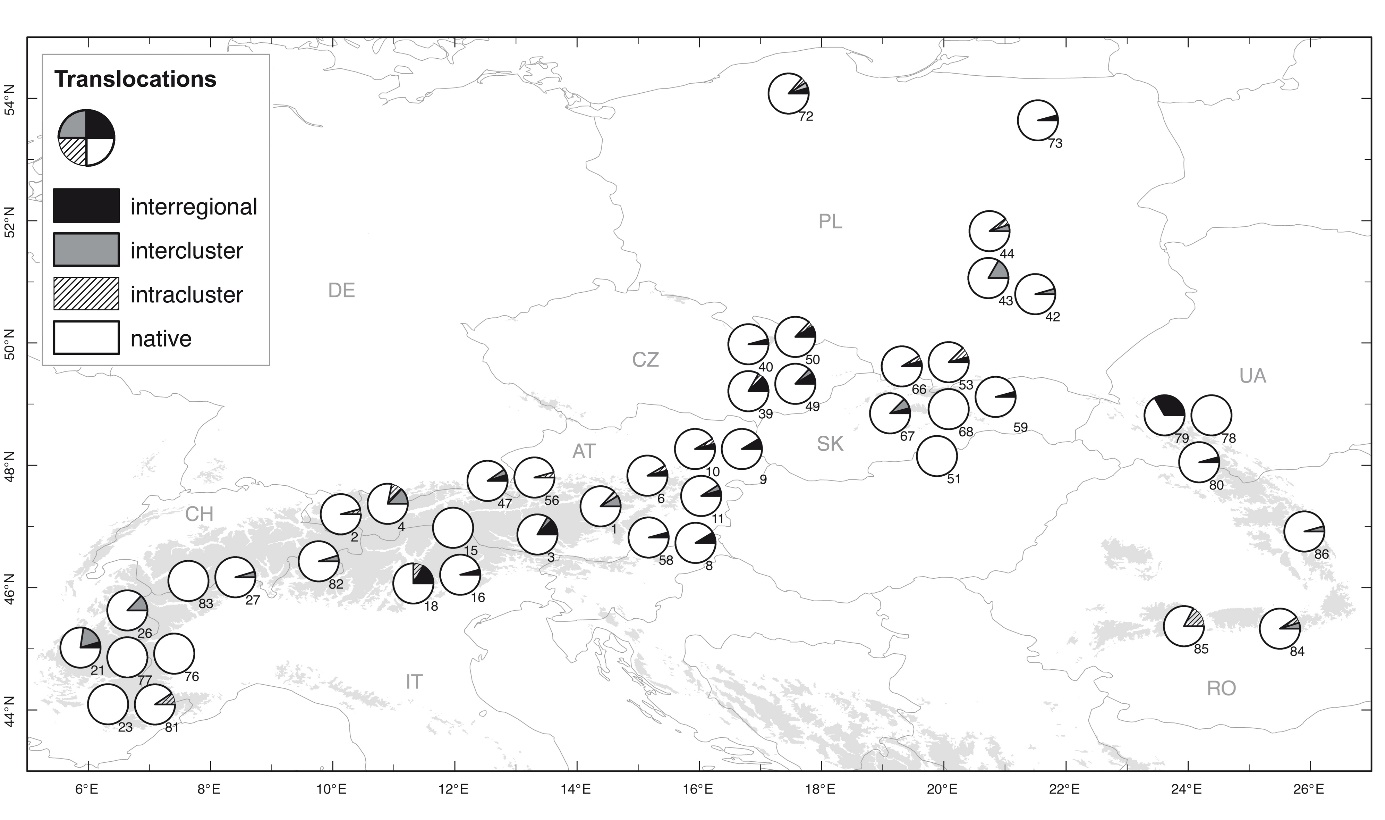


between-cluster group
within-cluster group
within-cluster
native
