# Supplementary material for: Within-Range Translocations and Their Consequences in European Larch
Source: PLoS One. 2015 May 22;10(5):e0127516. doi: 10.1371/journal.pone.0127516 (PMC4441476; doi:10.1371/journal.pone.0127516)
Supplement: S5 Fig — (DOCX) [file pone.0127516.s005.docx]

**S5 Fig.** Determining the number of populations with Structure using Evanno’s method

| **K** | **LnP(D)** | **L(K) sd** | **delta K** |
| --- | --- | --- | --- |
| 2 | -57237,39 | 2,13 | 369,26 |
| **3** | **-55646,24** | **2,17** | **530,14** |
| 4 | -55204,74 | 1335,02 | 0,19 |
| 5 | -55010,99 | 2805,84 | 0,35 |
| 6 | -53846,35 | 186,71 | 4,16 |
| **7** | **-53458,51** | **18,51** | **9,28** |
| 8 | -53242,43 | 117,09 | 0,63 |
| 9 | -53100,08 | 97,94 | 0,45 |

**References**

1. Earl D and vonHoldt B (2012) STRUCTURE HARVESTER: a website and program for visualizing STRUCTURE output and implementing the Evanno method. Conservation Genetics Resources 4: 359-361.

2. Evanno G, Regnaut S and Goudet J (2005) Detecting the number of clusters of individuals using the software STRUCTURE: a simulation study. Molecular Ecology 14: 2611–2620.
