# Supplementary material for: Within-Range Translocations and Their Consequences in European Larch
Source: PLoS One. 2015 May 22;10(5):e0127516. doi: 10.1371/journal.pone.0127516 (PMC4441476; doi:10.1371/journal.pone.0127516)
Supplement: S6 Fig — (DOCX) [file pone.0127516.s006.docx]

**S6 Fig.** Results of Principle Coordinate Analyses ([1](#_ENREF_1)) before (a) and after (b) systematic translocation removal.

|  | Structure cluster 1 |  | Structure cluster 3 |  | Structure cluster 5 |  | Structure cluster 7 |
| --- | --- | --- | --- | --- | --- | --- | --- |
|  |  |  |  |  |  |  |  |
|  | Structure cluster 2 |  | Structure cluster 4 |  | Structure cluster 6 |  |  |

| **Eigen Values by Axis and Sample Eigen Vectors before translocation removal** | | |  |  |  |  |
| --- | --- | --- | --- | --- | --- | --- |
| **Axis No.** | **1** | **2** | **3** | **4** | **5** | **6** |
| **EigenValue** | 0.039 | 0.025 | 0.014 | 0.013 | 0.010 | 0.007 |
| **81** | 0.047 | -0.035 | -0.002 | -0.006 | -0.009 | 0.004 |
| **77** | 0.045 | -0.035 | 0.006 | -0.022 | -0.020 | 0.007 |
| **26** | 0.040 | -0.039 | 0.005 | -0.009 | -0.012 | -0.001 |
| **76** | 0.039 | -0.046 | 0.007 | -0.031 | -0.013 | 0.000 |
| **21** | 0.034 | -0.033 | 0.003 | -0.019 | -0.009 | -0.002 |
| **23** | 0.036 | -0.027 | 0.001 | -0.006 | -0.011 | 0.000 |
| **83** | 0.036 | -0.033 | 0.032 | 0.025 | 0.022 | 0.002 |
| **27** | 0.034 | -0.043 | 0.007 | 0.057 | 0.047 | -0.017 |
| **82** | 0.040 | 0.009 | -0.010 | -0.004 | -0.001 | 0.023 |
| **2** | 0.045 | 0.030 | -0.035 | -0.001 | 0.019 | 0.007 |
| **4** | 0.026 | 0.035 | -0.020 | -0.020 | 0.007 | -0.004 |
| **18** | 0.022 | 0.025 | -0.012 | -0.007 | 0.008 | 0.001 |
| **16** | 0.005 | 0.018 | -0.018 | -0.013 | 0.003 | 0.002 |
| **15** | 0.020 | 0.022 | -0.008 | 0.010 | 0.007 | -0.005 |
| **47** | -0.004 | 0.021 | 0.048 | 0.005 | -0.016 | 0.004 |
| **56** | 0.016 | 0.024 | 0.020 | 0.022 | 0.014 | 0.010 |
| **3** | 0.001 | 0.020 | 0.020 | 0.008 | -0.008 | -0.005 |
| **1** | 0.003 | 0.017 | 0.028 | 0.013 | 0.008 | 0.017 |
| **58** | 0.005 | 0.024 | 0.002 | -0.004 | 0.013 | 0.004 |
| **6** | 0.008 | 0.023 | 0.009 | 0.000 | -0.010 | -0.029 |
| **8** | -0.006 | 0.027 | 0.021 | -0.012 | -0.011 | -0.013 |
| **9** | -0.002 | 0.028 | 0.013 | 0.005 | -0.012 | -0.016 |
| **10** | 0.000 | 0.029 | 0.025 | 0.001 | -0.015 | -0.013 |
| **11** | 0.003 | 0.023 | 0.014 | -0.003 | -0.013 | -0.009 |
| **79** | -0.001 | 0.029 | 0.020 | -0.002 | -0.002 | 0.008 |
| **39** | -0.018 | 0.007 | -0.015 | 0.015 | -0.016 | 0.010 |
| **40** | -0.024 | -0.007 | -0.010 | 0.012 | -0.021 | 0.008 |
| **49** | -0.033 | -0.012 | -0.009 | 0.014 | -0.011 | 0.008 |
| **50** | -0.033 | -0.012 | -0.013 | 0.017 | -0.023 | 0.010 |
| **72** | -0.025 | -0.006 | -0.009 | 0.030 | -0.012 | 0.020 |
| **66** | -0.031 | -0.010 | -0.009 | 0.009 | -0.008 | 0.007 |
| **67** | -0.039 | -0.022 | -0.013 | 0.001 | 0.002 | -0.013 |
| **68** | -0.033 | -0.014 | -0.013 | 0.007 | -0.011 | -0.002 |
| **51** | -0.008 | 0.002 | -0.020 | 0.006 | 0.007 | 0.008 |
| **53** | -0.029 | -0.014 | -0.011 | -0.002 | 0.008 | -0.001 |
| **59** | -0.018 | -0.014 | -0.014 | -0.001 | -0.003 | -0.013 |
| **78** | 0.026 | 0.018 | -0.016 | -0.006 | 0.007 | 0.002 |
| **80** | -0.015 | 0.004 | -0.011 | 0.013 | -0.008 | 0.008 |
| **42** | -0.022 | 0.009 | -0.010 | 0.006 | -0.001 | -0.002 |
| **43** | -0.031 | -0.012 | -0.008 | -0.004 | 0.004 | -0.012 |
| **44** | -0.032 | -0.019 | -0.013 | 0.006 | 0.000 | -0.028 |
| **86** | -0.055 | -0.010 | 0.023 | -0.030 | 0.029 | 0.039 |
| **84** | -0.052 | -0.013 | 0.020 | -0.032 | 0.029 | -0.008 |
| **85** | -0.051 | -0.013 | 0.005 | -0.034 | 0.024 | -0.010 |
| **73** | 0.029 | 0.029 | -0.030 | -0.016 | 0.018 | -0.004 |

| **Eigen Values by Axis and Sample Eigen Vectors after translocation removal** | | |  |  |  |  |
| --- | --- | --- | --- | --- | --- | --- |
| **Axis No.** | **1** | **2** | **3** | **4** | **5** | **6** |
| **EigenValue** | 0.047 | 0.029 | 0.016 | 0.014 | 0.011 | 0.008 |
| **81** | 0.044 | -0.040 | 0.002 | -0.007 | -0.008 | 0.005 |
| **77** | 0.043 | -0.039 | 0.018 | -0.012 | -0.019 | 0.012 |
| **26** | 0.038 | -0.044 | 0.009 | -0.005 | -0.012 | -0.003 |
| **76** | 0.036 | -0.050 | 0.030 | -0.014 | -0.016 | 0.002 |
| **21** | 0.035 | -0.037 | 0.017 | -0.010 | -0.010 | 0.000 |
| **23** | 0.043 | -0.042 | 0.009 | 0.001 | -0.015 | 0.004 |
| **83** | 0.035 | -0.033 | 0.000 | 0.043 | 0.017 | 0.001 |
| **27** | 0.031 | -0.044 | -0.035 | 0.047 | 0.049 | -0.028 |
| **82** | 0.041 | 0.006 | -0.008 | -0.019 | 0.007 | 0.025 |
| **2** | 0.046 | 0.022 | -0.024 | -0.034 | 0.029 | 0.005 |
| **4** | 0.030 | 0.032 | 0.002 | -0.035 | 0.015 | -0.003 |
| **18** | 0.038 | 0.030 | -0.006 | -0.021 | 0.016 | 0.002 |
| **16** | 0.018 | 0.032 | 0.001 | -0.032 | 0.008 | -0.001 |
| **15** | 0.032 | 0.020 | -0.016 | -0.006 | 0.012 | -0.007 |
| **47** | 0.006 | 0.029 | 0.022 | 0.042 | -0.026 | 0.008 |
| **56** | 0.021 | 0.025 | -0.010 | 0.027 | 0.015 | 0.014 |
| **3** | 0.010 | 0.032 | 0.001 | 0.023 | -0.009 | 0.004 |
| **1** | 0.013 | 0.025 | 0.000 | 0.029 | 0.003 | 0.021 |
| **58** | 0.012 | 0.030 | 0.001 | -0.004 | 0.015 | 0.011 |
| **6** | 0.015 | 0.029 | 0.003 | 0.002 | -0.012 | -0.029 |
| **8** | 0.004 | 0.037 | 0.024 | 0.010 | -0.020 | -0.015 |
| **9** | 0.005 | 0.034 | 0.000 | 0.003 | -0.017 | -0.028 |
| **10** | 0.009 | 0.037 | 0.007 | 0.014 | -0.019 | -0.006 |
| **11** | 0.014 | 0.032 | 0.011 | 0.004 | -0.014 | -0.016 |
| **39** | -0.028 | -0.002 | -0.032 | -0.001 | -0.016 | 0.018 |
| **40** | -0.030 | -0.002 | -0.028 | -0.002 | -0.015 | 0.012 |
| **49** | -0.043 | -0.006 | -0.017 | 0.000 | -0.007 | 0.010 |
| **50** | -0.046 | -0.008 | -0.031 | -0.004 | -0.019 | 0.011 |
| **72** | -0.031 | -0.002 | -0.035 | 0.008 | -0.008 | 0.018 |
| **66** | -0.045 | -0.007 | -0.013 | -0.004 | -0.007 | 0.004 |
| **67** | -0.048 | -0.019 | -0.006 | -0.010 | 0.005 | -0.014 |
| **68** | -0.042 | -0.013 | -0.012 | -0.008 | -0.009 | -0.004 |
| **53** | -0.035 | -0.009 | -0.002 | -0.010 | 0.010 | -0.001 |
| **59** | -0.032 | -0.020 | -0.011 | -0.006 | -0.009 | -0.020 |
| **43** | -0.038 | -0.014 | 0.005 | -0.011 | 0.004 | -0.011 |
| **44** | -0.038 | -0.016 | -0.006 | -0.007 | 0.003 | -0.027 |
| **86** | -0.054 | 0.002 | 0.045 | 0.009 | 0.026 | 0.034 |
| **84** | -0.054 | -0.003 | 0.046 | 0.004 | 0.026 | -0.005 |
| **85** | -0.055 | -0.003 | 0.040 | -0.010 | 0.024 | -0.004 |

**Reference**

1. Peakall ROD, Smouse PE. GENEALEX 6: genetic analysis in Excel. Population genetic software for teaching and research. Molecular Ecology Notes. 2006;6(1):288-95.
