# Supplementary material for: Within-Range Translocations and Their Consequences in European Larch
Source: PLoS One. 2015 May 22;10(5):e0127516. doi: 10.1371/journal.pone.0127516 (PMC4441476; doi:10.1371/journal.pone.0127516)
Supplement: S7 Fig — (DOCX) [file pone.0127516.s007.docx]

**S7 Fig.** Comparison of Geneclass and Structure results


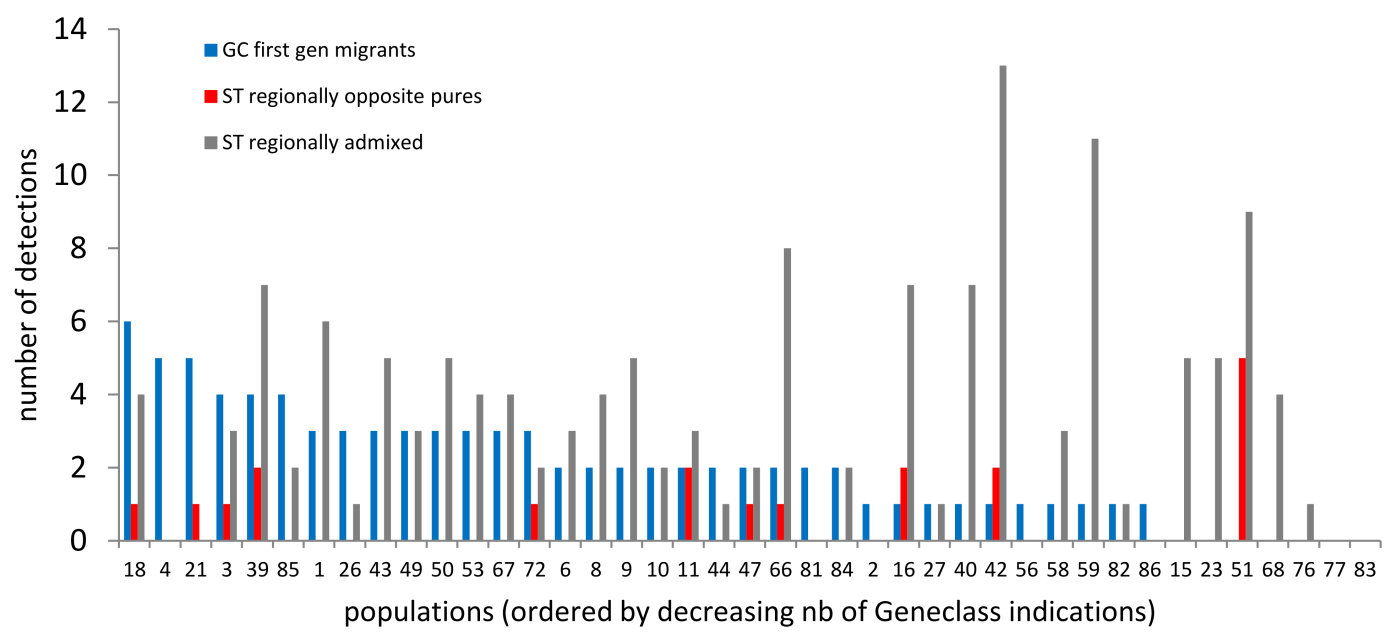


ST: admixed individuals

ST: purebred individuals

GC: recent migrants

When the number of recent migrants (detected with Geneclass) decreases, the number of cases of admixture detected by Structure increases.
