# Supplementary material for: Within-Range Translocations and Their Consequences in European Larch
Source: PLoS One. 2015 May 22;10(5):e0127516. doi: 10.1371/journal.pone.0127516 (PMC4441476; doi:10.1371/journal.pone.0127516)
Supplement: S1 Table — (DOCX) [file pone.0127516.s009.docx]

**S1 Table** Sampling information of the studied *Larix decidua* populations

| Pop ID | Lat dd | Lon dd | Alt | Provenance | Country | Trial location | Nb ind. studied for nSSR | Nb ind. studied for mt |
| --- | --- | --- | --- | --- | --- | --- | --- | --- |
| 1 | 47.33 | 14.53 | 1350 | Moederbrugg | AT | Münden | 23 | 8 |
| 2 | 47.20 | 10.67 | 1100 | Schoenwies | AT | Münden | 24 | 8 |
| 3 | 46.87 | 13.35 | 900 | Muehldorf | AT | Riedesel | 24 | 8 |
| 4 | 47.37 | 10.90 | 1150 | Fernpass | AT | Münden | 23 | 8 |
| 6 | 47.83 | 15.15 | 800 | Langau 45 | AT | Münden | 24 | 7 |
| 8 | 47.63 | 15.77 | 1200 | Semmering | AT | Riedesel | 23 | 10 |
| 9 | 48.05 | 15.93 | 610 | Lammerau | AT | Münden | 23 | 9 |
| 10 | 48.12 | 15.93 | 560 | Neulengbach | AT | Winnefeld | 24 | 9 |
| 11 | 47.50 | 16.03 | 1000 | Wechselgebiet | AT | Münden | 22 | 9 |
| 15 | 46.98 | 11.97 | 1100 | Bruneck | IT | Münden | 20 | 10 |
| 16 | 46.32 | 11.45 | 1200 | Cavalese | IT | Riedesel | 24 | 10 |
| 18 | 46.07 | 11.32 | 600 | Tenna | IT | Münden, Riedesel | 24 | 7 |
| 21 | 45.02 | 6.93 | 1900 | Pragelato | IT | Riedesel | 22 | 9 |
| 23 | 44.78 | 6.90 | 1560 | Embrun, Aiguilles | FR | Riedesel | 21 | 8 |
| 26 | 44.87 | 6.65 | 1400 | Briancon, de Villard | FR | Riedesel | 23 | 8 |
| 27 | 46.17 | 7.83 | 1550 | St Niklaus | CH | Riedesel | 22 | 8 |
| 39 | 49.83 | 16.97 | 400 | Zabreh-Dubicko | CZ | Riedesel | 24 | 8 |
| 40 | 49.98 | 16.90 | 480 | Ruda nad Moravou | CZ | Riedesel | 24 | 9 |
| 42 | 50.80 | 21.10 | 347 | Gora Chelmova | PL | Sellhorn | 23 | 9 |
| 43 | 51.07 | 20.73 | 330 | Blizyn | PL | Sellhorn | 18 | 8 |
| 44 | 51.83 | 20.75 | 180 | Mala Wies | PL | Riedesel | 19 | 8 |
| 47 | 47.47 | 13.10 | 830 | Bluehnbachtal | AT | Spießingsho | 21 | 8 |
| 49 | 50.05 | 17.55 | 550 | Krnov | CZ | Riedesel | 23 | 9 |
| 50 | 50.10 | 17.57 | 450 | Krnov | CZ | Münden | 23 | 9 |
| 51 | 49.00 | 19.88 | 800 | Cierny Vah | CZ | Münden | 20 | 7 |
| 53 | 49.13 | 20.18 | 1200 | Smokovec | CZ | Riedesel, Spießingshol | 24 | 12 |
| 56 | 47.80 | 13.30 | 700 | Fuschlsee | AT | Oerrel | 23 | 8 |
| 58 | 46.82 | 15.17 | 800 | Deutschlandsberg | AT | Münden | 24 | 9 |
| 59 | 49.12 | 20.82 | 830 | Brezovicka | CZ | Münden | 24 | 10 |
| 66 | 49.00 | 19.88 | 750 | Cierny | CZ | Münden | 23 | 9 |
| 67 | 48.85 | 19.12 | 850 | Stare Hory | CZ | Münden, Oerrel | 24 | 9 |
| 68 | 48.92 | 20.08 | 1400 | Liptovska | CZ | Rotenburg | 25 | 8 |
| 76 | 44.92 | 6.72 | 1500 | Briançon, Montgenèvre | FR | *in situ* | 24 | 8 |
| 77 | 44.86 | 6.64 | 1500 | Briançon, de Villard | FR | *in situ* | 25 | 8 |
| 81 | 44.09 | 7.09 | 1070 | Vallée de la Tinée | FR | *in situ* | 21 | 7 |
| 82 | 46.43 | 9.77 | 2000 | Sils Maria | CH | *in situ* | 21 | 8 |
| 83 | 46.11 | 7.64 | 1800 | Zinal | CH | *in situ* | 21 | 6 |
| 84 | 45.33 | 25.50 | 1500 | Sinaia Forest District | RO | *in situ* | 21 | 9 |
| 85 | 45.37 | 23.93 | 1000 | Voineasa Forest District | RO | *in situ* | 23 | 8 |
| 86 | 46.92 | 25.90 | 1500 | Bicaz | RO | *in situ* | 24 | 6 |
| 72* | 54.08 | 17.46 | 184 | Rekovo | PL | *in situ* | 22 | 10 |
| 73* | 53.64 | 21.54 | 132 | Ruciane-Nida | PL | *in situ* | 24 | 9 |
| 78* | 48.65 | 24.38 | 423 | Solotwyn | UA | *in situ* | 24 | - |
| 79* | 48.42 | 24.02 | 1100 | Brusturjany | UA | *in situ* | 24 | 8 |
| 80* | 48.05 | 24.18 | 500 | Rahiv | UA | *in situ* | 24 | - |

* sampled outside the native range
