# Supplementary material for: Within-Range Translocations and Their Consequences in European Larch
Source: PLoS One. 2015 May 22;10(5):e0127516. doi: 10.1371/journal.pone.0127516 (PMC4441476; doi:10.1371/journal.pone.0127516)
Supplement: S2 Table — (DOCX) [file pone.0127516.s010.docx]

**S2 Table** Nucleotide and minisatellite variation of 23 mitochondrial haplotypes of *L. decidua* and *L. sibirica* (H23 only). Nucleotide position according to alignment position is provided for substitutions. Copy number of atpA-minisatellite for motifs A and B are provided.

|  |  | **atpA consensus position** | | | | | **UBC460 consensus position** | | | |
| --- | --- | --- | --- | --- | --- | --- | --- | --- | --- | --- |
| **Haplotype Code** | **Count** | **672** | **780** | **1207** | **A(n)^1^** | **B(n)^2^** | **34** | **123** | **644** | **734** |
| H1 | 1 | G | G | G | 2 | 14 | G | C | A | C |
| H2 | 2 | G | G | G | 2 | 10 | G | C | A | C |
| H3 | 9 | G | G | G | 2 | 4 | G | C | A | C |
| H4 | 2 | G | G | G | 2 | 3 |  | C | A | C |
| H5 | 7 | G | G | G | 2 | 3 | G | C | C | C |
| H6 | 2 | G | G | G | 2 | 2 | G | C | C | C |
| H7 | 1 | G | G | G | 1 | 4 | G | C | C | C |
| H8 | 2 | G | G | G | 1 | 1 | G | C | A | A |
| H9 | 56 | G | G | G | 1 | 1 | G | C | A | C |
| H10 | 53 | G | G | G | 1 | 1 | G | C | C | C |
| H11 | 1 | G | G | G | 0 | 26 | G | C | A | C |
| H12 | 1 | G | G | G | 0 | 24 | G | C | A | C |
| H13 | 1 | G | G | G | 0 | 21 | G | C | C | C |
| H14 | 4 | G | G | G | 0 | 16 | G | C | A | C |
| H15 | 1 | G | G | G | 0 | 14 | G | C | A | C |
| H16 | 65 | G | G | G | 0 | 1 | G | C | A | A |
| H17 | 1 | G | G | G | 0 | 1 | G | C | A | C |
| H18 | 156 | G | G | G | 0 | 1 | T | C | A | A |
| H19 | 3 | G | G | T | 1 | 2 | G | C | A | C |
| H20 | 2 | G | G | T | 1 | 1 | G | C | C | C |
| H21 | 4 | G | T | G | 1 | 0 | G | A | A | C |
| H22 | 11 | G | T | G | 0 | 1 | T | C | A | A |
| H23 | 8 | T | G | G | 0 | 1 | G | C | A | C |

^1^sequence of minisatellite motif A: GGAAAAGCTTGCTTGACATTAGGAGAGCAA

^2^sequence of minisatellite motif B: GGAAAAGCTTGCTTGACCGTAAGGGAGAGCAA
