# Supplementary material for: Within-Range Translocations and Their Consequences in European Larch
Source: PLoS One. 2015 May 22;10(5):e0127516. doi: 10.1371/journal.pone.0127516 (PMC4441476; doi:10.1371/journal.pone.0127516)
Supplement: S3 Table — (DOCX) [file pone.0127516.s011.docx]

**S3 Table** False positives detection by different simulations (group 1: cluster 1-4, group 2: cluster 5-7).

|  | Threshold: | 0.875 | 0.9375 | 0.875 | 0.9375 |
| --- | --- | --- | --- | --- | --- |
|  | Proportion of genotypes misassigned to: | purebreds  opposite group | purebreds  opposite group | admixed | admixed |
| Random mating within | group 1 | 0 | - | 0.133 | - |
|  | group 2 | 0 | - | 0.123 | - |
|  | cluster 1 | 0 | 0 | 0.013 | 0.008 |
|  | cluster 2 | 0 | 0 | 0.024 | 0.008 |
|  | cluster 3 | 0 | 0 | 0.023 | 0.012 |
|  | cluster 4 | 0 | 0 | 0.046 | 0.038 |
|  | cluster 5 | 0 | 0 | 0.054 | 0.031 |
|  | cluster 6 | 0 | 0 | 0.111 | 0.037 |
|  | cluster 7 | 0 | 0 | 0.059 | 0.028 |
