# Supplementary material for: Within-Range Translocations and Their Consequences in European Larch
Source: PLoS One. 2015 May 22;10(5):e0127516. doi: 10.1371/journal.pone.0127516 (PMC4441476; doi:10.1371/journal.pone.0127516)
Supplement: S4 Table — (DOCX) [file pone.0127516.s012.docx]

**S4 Table** *F*_IS_ before and after systematic translocation removal

| **POP*** | **g81** | **f81** | **g77** | **f77** | **g26** | **f26** | **g76** | **f76** | **g21** | **f21** | **g23** | **f23** | **g83** | **f83** | **g27** | **f27** | **g82** | **f82** |
| --- | --- | --- | --- | --- | --- | --- | --- | --- | --- | --- | --- | --- | --- | --- | --- | --- | --- | --- |
| ***F*_IS_ per population** |  |  |  |  |  |  |  |  |  |  |  |  |  |  |  |  |  |  |
| bcLK25 | -0.054 | -0.057 | 0.002 | 0.002 | 0.156 | 0.199 | 0.007 | 0.007 | 0.12 | -0.019 | 0.02 | 0 | 0.156 | 0.156 | -0.062 | -0.143 | -0.078 | -0.073 |
| bcLK21 | -0.01 | -0.091 | 0.086 | 0.086 | -0.097 | -0.172 | 0.095 | 0.104 | 0.115 | 0.099 | 0.016 | -0.09 | -0.074 | -0.074 | 0.078 | 0.012 | -0.048 | -0.085 |
| bcLK22 | -0.191 | -0.218 | 0.1 | 0.1 | 0.126 | 0.094 | 0.019 | -0.005 | -0.055 | -0.178 | 0.067 | 0.027 | -0.003 | -0.003 | -0.217 | -0.313 | -0.121 | -0.115 |
| Ld30 | 0.222 | 0.259 | -0.091 | -0.091 | -0.066 | -0.186 | -0.019 | 0.001 | 0.17 | 0.026 | -0.02 | 0.018 | -0.221 | -0.221 | -0.054 | -0.188 | 0.25 | 0.224 |
| Ld31 | -0.12 | -0.091 | -0.25 | -0.25 | -0.108 | -0.171 | -0.011 | -0.007 | 0.185 | 0.096 | -0.013 | -0.022 | 0.068 | 0.068 | -0.228 | -0.255 | -0.018 | 0.009 |
| Ld50 | 0.321 | 0.278 | 0.193 | 0.193 | 0.018 | -0.266 | 0.016 | -0.041 | -0.011 | -0.215 | 0.018 | 0.004 | 0.134 | 0.134 | 0.159 | 0.126 | 0.02 | -0.098 |
| bcLK18 | -0.019 | 0.012 | -0.209 | -0.209 | -0.202 | -0.259 | -0.048 | -0.037 | 0.121 | 0.226 | 0.039 | -0.04 | 0.059 | 0.059 | -0.143 | -0.222 | 0.317 | 0.299 |
| Ld101 | -0.054 | -0.057 | 0.098 | 0.098 | 0.181 | 0 | 0.003 | 0.017 | 0.101 | -0.032 | -0.054 | -0.008 | 0.056 | 0.056 | 0.068 | 0.12 | -0.026 | -0.025 |
| Ld42 | 0.247 | 0.284 | 0.241 | 0.241 | 0.117 | -0.179 | -0.066 | -0.029 | 0.164 | -0.093 | 0.27 | 0.259 | 0.003 | 0.003 | -0.082 | -0.063 | 0.062 | -0.157 |
| Ld56 | -0.087 | -0.089 | -0.124 | -0.124 | -0.058 | -0.125 | 0.068 | 0.083 | 0.048 | 0.057 | 0.095 | 0.152 | -0.166 | -0.166 | -0.123 | -0.173 | 0.021 | 0.019 |
| Ld58 | 0.246 | 0.215 | -0.075 | -0.075 | 0.027 | -0.085 | -0.002 | 0.012 | 0.169 | 0.121 | 0.246 | 0.197 | 0.049 | 0.049 | 0.077 | 0.13 | 0.127 | 0.164 |
| bcLK26 | -0.014 | -0.016 | -0.058 | -0.058 | 0.113 | 0.097 | 0.043 | 0.048 | 0.107 | 0.086 | 0.183 | 0.167 | 0.162 | 0.162 | 0.086 | 0.113 | 0.021 | 0.025 |
| Ld45 | 0.021 | -0.053 | 0.165 | 0.165 | 0.162 | 0.17 | -0.082 | -0.073 | 0.194 | -0.009 | 0.045 | 0.021 | 0.188 | 0.188 | -0.004 | -0.005 | 0.045 | 0.066 |
| All | 0.034 | 0.02 | -0.002 | -0.002 | 0.024 | -0.062 | 0.005 | 0.01 | 0.111 | 0.019 | 0.07 | 0.05 | 0.029 | 0.029 | -0.032 | -0.066 | 0.046 | 0.029 |

| **POP*** | **g2** | **f2** | **g4** | **f4** | **g18** | **f18** | **g16** | **f16** | **g15** | **f15** | **g47** | **f47** | **g56** | **f56** | **g3** | **f3** | **g1** | **f1** |
| --- | --- | --- | --- | --- | --- | --- | --- | --- | --- | --- | --- | --- | --- | --- | --- | --- | --- | --- |
| ***F*_IS_ per population** |  |  |  |  |  |  |  |  |  |  |  |  |  |  |  |  |  |  |
| bcLK25 | 0.055 | -0.003 | 0.072 | -0.069 | 0.045 | 0.039 | -0.059 | -0.114 | -0.079 | -0.04 | 0.079 | 0.057 | 0.143 | 0.16 | 0.035 | -0.036 | 0.046 | 0.027 |
| bcLK21 | -0.064 | -0.095 | -0.03 | -0.094 | 0.062 | 0.018 | 0.14 | -0.049 | -0.024 | -0.006 | 0.169 | 0.044 | 0.073 | 0.091 | 0.034 | 0.025 | -0.026 | -0.106 |
| bcLK22 | -0.205 | -0.195 | -0.01 | -0.122 | -0.035 | -0.032 | 0.033 | 0.083 | 0.011 | 0.04 | -0.061 | -0.06 | 0.006 | 0.012 | 0.227 | 0.299 | -0.198 | -0.348 |
| Ld30 | -0.021 | 0 | 0.031 | -0.205 | -0.026 | -0.211 | 0.013 | 0.061 | -0.144 | -0.19 | 0.213 | 0.345 | -0.062 | -0.077 | 0.083 | 0.015 | -0.073 | -0.092 |
| Ld31 | 0.214 | 0.193 | -0.122 | -0.193 | -0.147 | -0.199 | -0.03 | 0.006 | -0.032 | -0.08 | 0.021 | -0.014 | -0.025 | 0.005 | 0.107 | 0.128 | 0.065 | 0.112 |
| Ld50 | -0.096 | -0.075 | 0.052 | 0.018 | 0.071 | 0.086 | -0.026 | -0.263 | -0.224 | -0.382 | 0.034 | -0.003 | -0.003 | -0.02 | 0.18 | 0.02 | 0.132 | 0.081 |
| bcLK18 | 0.019 | -0.061 | -0.078 | -0.051 | 0.012 | -0.078 | 0.069 | -0.034 | 0.089 | -0.026 | 0.075 | 0.111 | 0.079 | 0.056 | 0.261 | 0.164 | 0.133 | 0.125 |
| Ld101 | -0.045 | -0.066 | -0.077 | -0.067 | 0.582 | 0.458 | -0.177 | -0.102 | 0.034 | 0.028 | -0.053 | -0.067 | -0.116 | -0.096 | 0.29 | -0.014 | -0.054 | -0.054 |
| Ld42 | -0.136 | -0.151 | 0.121 | 0.026 | 0.223 | 0.39 | 0.188 | -0.077 | -0.179 | -0.151 | 0.145 | 0.034 | -0.143 | -0.151 | 0.096 | -0.008 | 0.031 | -0.02 |
| Ld56 | -0.085 | -0.119 | -0.114 | -0.167 | -0.007 | -0.158 | 0.064 | 0 | 0.015 | 0.04 | -0.148 | -0.071 | -0.156 | -0.157 | 0.023 | -0.032 | 0.156 | 0.081 |
| Ld58 | 0.093 | 0.031 | 0.085 | 0.072 | 0.084 | 0.082 | 0.076 | 0.109 | -0.042 | -0.021 | 0.282 | 0.304 | -0.007 | -0.073 | -0.032 | -0.024 | 0.109 | 0.106 |
| bcLK26 | -0.056 | -0.052 | 0.201 | 0.182 | 0.151 | -0.009 | 0.093 | -0.047 | -0.167 | -0.189 | -0.064 | -0.106 | -0.016 | -0.024 | 0.028 | 0.05 | 0.073 | 0.054 |
| Ld45 | -0.177 | -0.164 | 0.019 | 0.072 | 0.054 | -0.091 | 0.083 | -0.06 | 0.063 | 0.107 | -0.09 | -0.221 | -0.102 | -0.083 | 0.428 | 0.434 | -0.008 | 0.091 |
| All | -0.032 | -0.053 | 0.022 | -0.03 | 0.058 | -0.007 | 0.042 | -0.03 | -0.051 | -0.066 | 0.056 | 0.032 | -0.011 | -0.014 | 0.125 | 0.089 | 0.036 | 0.014 |

* g: global sample, f: final sample after removal; *F*_IS_ was not recalculated if >50% of the individuals had been removed (populations 42, 51)

Table S3 continued

| **POP** | **g58** | **f58** | **g6** | **f6** | **g8** | **f8** | **g9** | **f9** | **g10** | **f10** | **g11** | **f11** | **g79** | **g39** | **f39** | **g40** | **f40** |
| --- | --- | --- | --- | --- | --- | --- | --- | --- | --- | --- | --- | --- | --- | --- | --- | --- | --- |
| ***F*_IS_ per population** |  |  |  |  |  |  |  |  |  |  |  |  |  |  |  |  |  |
| bcLK25 | -0.006 | -0.032 | -0.02 | -0.038 | 0.065 | -0.014 | -0.048 | -0.036 | -0.083 | -0.164 | 0.08 | 0.016 | 0.011 | -0.087 | -0.025 | 0.011 | -0.082 |
| bcLK21 | 0.031 | 0.016 | 0.064 | 0.017 | -0.146 | -0.168 | -0.184 | -0.195 | 0.138 | 0.084 | 0.243 | 0.327 | 0.282 | 0.038 | -0.036 | -0.16 | -0.117 |
| bcLK22 | -0.11 | -0.106 | -0.008 | -0.027 | -0.072 | -0.059 | -0.051 | -0.046 | 0.254 | 0.297 | -0.017 | -0.109 | -0.072 | -0.034 | -0.162 | 0.09 | 0.036 |
| Ld30 | -0.035 | -0.056 | 0.07 | 0.094 | 0.025 | 0.053 | -0.064 | -0.148 | 0.246 | 0.255 | 0.036 | -0.061 | 0.254 | 0.062 | 0.26 | 0.1 | 0.05 |
| Ld31 | -0.008 | 0.04 | 0.085 | 0.027 | 0.049 | -0.021 | -0.044 | -0.118 | 0.179 | 0.133 | 0.111 | 0.262 | -0.002 | -0.129 | -0.309 | 0.111 | 0.082 |
| Ld50 | 0.065 | 0.085 | 0.025 | 0.007 | -0.184 | -0.152 | 0.088 | -0.013 | 0.009 | 0.043 | 0.087 | 0.202 | 0.14 | 0.169 | 0.12 | -0.064 | -0.113 |
| bcLK18 | -0.235 | -0.226 | 0.134 | 0.203 | 0.135 | 0.078 | 0.203 | 0.084 | 0.007 | 0.007 | -0.023 | -0.054 | 0.12 | 0.018 | -0.108 | 0.148 | 0.183 |
| Ld101 | 0.363 | 0.431 | -0.04 | -0.027 | -0.023 | 0 | -0.128 | -0.172 | -0.022 | -0.026 | 0.25 | 0.474 | 0.29 | 0.108 | 0.184 | 0.108 | 0.138 |
| Ld42 | 0.142 | -0.109 | 0.023 | -0.027 | 0.217 | 0.227 | 0.366 | 0.328 | 0.018 | -0.063 | 0.16 | 0.018 | -0.139 | 0.108 | -0.013 | -0.024 | -0.167 |
| Ld56 | -0.001 | -0.007 | -0.074 | -0.088 | -0.219 | -0.204 | 0.06 | -0.047 | -0.072 | -0.061 | 0.162 | 0.136 | 0.196 | 0.01 | -0.068 | 0.19 | 0.193 |
| Ld58 | -0.004 | 0.026 | -0.052 | -0.112 | 0.051 | 0.054 | 0.067 | -0.063 | -0.138 | -0.138 | 0.048 | -0.074 | 0.321 | 0.17 | 0.287 | 0.048 | 0.051 |
| bcLK26 | 0.203 | 0.26 | -0.042 | -0.079 | 0.035 | -0.057 | 0.032 | 0.074 | -0.048 | -0.058 | 0.037 | 0.089 | -0.051 | 0.448 | 0.516 | 0.208 | 0.154 |
| Ld45 | -0.204 | -0.195 | -0.009 | -0.026 | -0.037 | -0.071 | 0.229 | 0.118 | 0.287 | 0.296 | -0.008 | -0.135 | 0.025 | 0.02 | -0.091 | 0.041 | 0.002 |
| All | -0.001 | -0.002 | 0.015 | -0.005 | -0.006 | -0.03 | 0.038 | -0.019 | 0.064 | 0.052 | 0.079 | 0.053 | 0.1 | 0.073 | 0.047 | 0.065 | 0.036 |

| **POP** | **g49** | **f49** | **g50** | **f50** | **g72** | **F72** | **g66** | **f66** | **g67** | **f67** | **g68** | **f68** | **g51** | **g53** | **f53** | **g59** | **f59** |
| --- | --- | --- | --- | --- | --- | --- | --- | --- | --- | --- | --- | --- | --- | --- | --- | --- | --- |
| ***F*_IS_ per population** |  |  |  |  |  |  |  |  |  |  |  |  |  |  |  |  |  |
| bcLK25 | -0.153 | -0.156 | -0.086 | -0.039 | -0.011 | -0.062 | 0.109 | 0.036 | -0.065 | -0.078 | -0.12 | -0.133 | -0.042 | -0.047 | -0.015 | 0.02 | 0.023 |
| bcLK21 | 0.066 | -0.012 | 0.036 | -0.043 | 0.272 | 0.229 | -0.011 | 0.022 | -0.027 | -0.037 | 0.002 | 0.056 | -0.084 | -0.106 | -0.078 | 0.055 | 0.022 |
| bcLK22 | -0.098 | -0.082 | 0.003 | -0.021 | -0.038 | -0.106 | -0.073 | -0.139 | 0.148 | 0.224 | 0.048 | 0.082 | -0.006 | -0.011 | -0.07 | -0.07 | -0.068 |
| Ld30 | -0.121 | -0.129 | 0.077 | -0.021 | -0.072 | -0.114 | -0.175 | -0.148 | -0.082 | -0.103 | 0.075 | 0.021 | 0.029 | 0.228 | 0.27 | -0.07 | -0.134 |
| Ld31 | -0.094 | -0.095 | 0.045 | 0.092 | 0.11 | 0.13 | 0.19 | 0.044 | 0.061 | 0.067 | 0.051 | 0.081 | -0.021 | 0.062 | -0.071 | -0.006 | 0.08 |
| Ld50 | -0.004 | 0.025 | 0.058 | 0.015 | 0.173 | 0.16 | 0.225 | 0.366 | 0.031 | -0.012 | 0.199 | 0.199 | 0.011 | 0.189 | 0.223 | 0.1 | -0.099 |
| bcLK18 | 0.073 | 0.02 | 0.112 | 0.091 | 0.019 | 0.018 | -0.034 | -0.12 | 0.035 | -0.011 | 0.01 | 0.036 | -0.025 | -0.044 | 0.002 | -0.001 | -0.101 |
| Ld101 | 0.012 | 0.089 | 0.021 | -0.022 | 0.017 | 0.142 | -0.138 | -0.036 | 0.429 | 0.521 | -0.162 | -0.145 | -0.034 | 0.066 | 0.084 | 0.11 | 0.262 |
| Ld42 | 0.063 | -0.006 | 0.129 | 0.129 | -0.071 | 0.015 | 0.284 | 0.392 | 0.01 | -0.068 | 0.207 | 0.171 | -0.106 | 0.134 | 0.214 | 0.241 | 0.273 |
| Ld56 | 0.106 | 0.098 | -0.099 | -0.244 | 0.203 | 0.216 | 0.292 | 0.514 | 0.36 | 0.202 | 0.027 | 0.004 | 0.136 | 0.179 | 0.252 | 0.153 | 0.098 |
| Ld58 | 0.174 | 0.163 | 0.002 | 0.026 | 0.08 | 0.008 | 0.021 | 0.077 | 0.019 | -0.026 | -0.052 | -0.032 | 0.013 | 0.074 | 0.133 | 0.08 | -0.021 |
| bcLK26 | 0.554 | 0.524 | 0.299 | 0.293 | 0.411 | 0.479 | 0.345 | 0.568 | 0.114 | 0.005 | 0.232 | 0.277 | 0.084 | 0.08 | 0.05 | 0.19 | 0.282 |
| Ld45 | -0.193 | -0.291 | 0.146 | 0.269 | -0.093 | -0.13 | 0.108 | 0.2 | -0.026 | -0.011 | -0.012 | 0.03 | 0.094 | 0.011 | 0.121 | 0.181 | 0.287 |
| All | 0.037 | 0.016 | 0.058 | 0.043 | 0.088 | 0.077 | 0.098 | 0.149 | 0.071 | 0.04 | 0.046 | 0.059 | 0.009 | 0.062 | 0.085 | 0.073 | 0.063 |

* g: global sample, f: final sample after removal; *F*_IS_ was not recalculated if >50% of the individuals had been removed (populations 42, 51)

Table S3 continued

| **POP** | **g78** | **g80** | **g42** | **g43** | **f43** | **g44** | **f44** | **g86** | **f86** | **g84** | **f84** | **g85** | **f85** | **g73** |
| --- | --- | --- | --- | --- | --- | --- | --- | --- | --- | --- | --- | --- | --- | --- |
| **Fis per population** |  |  |  |  |  |  |  |  |  |  |  |  |  |  |
| bcLK25 | 0.004 | 0.102 | -0.075 | 0.087 | 0.06 | 0.029 | -0.058 | -0.069 | -0.07 | 0.004 | 0.015 | -0.006 | -0.028 | 0.013 |
| bcLK21 | 0.278 | 0.021 | 0.034 | 0.046 | 0.03 | -0.08 | -0.117 | -0.115 | -0.139 | 0.287 | 0.259 | -0.038 | -0.015 | 0.085 |
| bcLK22 | 0.001 | 0.121 | 0.093 | 0.111 | 0.058 | -0.01 | 0.005 | 0.061 | 0.07 | -0.128 | -0.146 | -0.041 | -0.092 | -0.045 |
| Ld30 | -0.042 | 0.153 | 0.021 | 0.155 | -0.174 | 0.279 | 0.375 | 0.204 | 0.239 | 0.185 | 0.353 | 0.192 | 0.148 | -0.062 |
| Ld31 | -0.143 | -0.062 | 0.064 | 0.049 | -0.052 | -0.073 | -0.06 | 0.302 | 0.258 | 0.253 | 0.259 | 0.217 | 0.127 | -0.04 |
| Ld50 | -0.044 | 0.12 | 0.233 | 0.155 | -0.025 | 0.042 | 0.002 | 0.125 | 0.141 | -0.107 | -0.094 | 0.099 | 0.131 | -0.087 |
| bcLK18 | 0.095 | 0.2 | -0.027 | 0.111 | 0.169 | -0.013 | -0.055 | 0.082 | 0.065 | 0.28 | 0.337 | 0.08 | 0.197 | -0.084 |
| Ld101 | -0.228 | 0.082 | 0.38 | 0.092 | -0.213 | -0.157 | -0.25 | -0.136 | -0.143 | 0.135 | 0 | -0.038 | 0.005 | 0.1 |
| Ld42 | -0.029 | -0.015 | 0.104 | 0.243 | 0.125 | -0.195 | -0.278 | 0.079 | 0.032 | -0.12 | -0.164 | 0.014 | 0.059 | 0.093 |
| Ld56 | -0.045 | 0.052 | 0.051 | 0.382 | 0.275 | 0.036 | 0.013 | 0.248 | 0.11 | 0.183 | 0.226 | 0.085 | 0.096 | 0.01 |
| Ld58 | 0.128 | 0.058 | -0.003 | 0.03 | -0.038 | -0.043 | -0.037 | 0.018 | -0.04 | -0.017 | -0.041 | 0.028 | 0.104 | 0.208 |
| bcLK26 | 0.026 | 0.283 | 0.154 | 0.111 | 0.241 | -0.045 | -0.051 | -0.118 | -0.115 | 0.331 | 0.212 | -0.015 | -0.021 | 0.016 |
| Ld45 | 0.006 | -0.017 | 0.041 | 0.195 | 0.223 | -0.056 | -0.098 | 0.068 | -0.162 | 0.432 | 0.424 | 0.207 | 0.137 | 0.121 |
| All | 0.007 | 0.089 | 0.068 | 0.134 | 0.06 | -0.015 | -0.033 | 0.056 | 0.03 | 0.137 | 0.139 | 0.062 | 0.062 | 0.023 |

* g: global sample, f: final sample after removal; *F*_IS_ was not recalculated if >50% of the individuals had been removed (populations 42, 51)
