# Supplementary material for: Within-Range Translocations and Their Consequences in European Larch
Source: PLoS One. 2015 May 22;10(5):e0127516. doi: 10.1371/journal.pone.0127516 (PMC4441476; doi:10.1371/journal.pone.0127516)
Supplement: S1 Text — (DOCX) [file pone.0127516.s013.docx]

**S1 Text Definitions**

Translocation is the human-mediated transfer of living organisms from one place to another. The three main classes of translocation distinguished in this paper are defined as follows:

-Introduction of an organism is the intentional or accidental dispersal by human agency of a living organism outside its historically known native range.

-Re-introduction of an organism is the intentional movement of an organism into a part of its native range from which it has disappeared or become extirpated in historic times as a result of human activities or natural catastrophe.

-Re-stocking is the intentional movement of an organism with the intention of building up the number of individuals of that species in a given part of its range.

IUCN (1987) IUCN position statement on the translocation of living organisms: introductions, re-introduction, and re-stocking. IUCN, Gland, Switzerland. <http://intranet.iucn.org/webfiles/doc/SSC/SSCwebsite/Policy_statements/IUCN_Position_Statement_on_Translocation_of_Living_Organisms.pdf>.
